# Supplementary material for: Phytochemical and biological investigations on Centranthus kellereri (Stoj., Stef. & T. Georgiev) Stoj. & Stef. and C. ruber (L.) DC. and their potential as new medicinal and ornamental plants
Source: PLoS One. 2023 Nov 7;18(11):e0293877. doi: 10.1371/journal.pone.0293877 (PMC10629660; doi:10.1371/journal.pone.0293877)
Supplement: S1 File — (DOCX) [file pone.0293877.s001.docx]

**Table S1** Data used for the 2018 experiment.

| Location | Plantpart | Furfural | 3-Methylbutanoic acid (isovaleric acid) | 3-Methylpentanoic acid (3-methylvareric acid) | Benzene acetaldehyde (hyacinthin) | Borneol | Other (aldechide, alchohol, ethers) | Monoterpenoid | Methyl-branched fatty acid |
| --- | --- | --- | --- | --- | --- | --- | --- | --- | --- |
| Bansko | Abovground | 4.783 | 61.692 | 6.538 | 3.989 | 14.908 | 11.574 | 18.727 | 68.230 |
| Bansko | Abovground | 5.055 | 63.195 | 5.909 | 3.581 | 13.755 | 11.597 | 17.792 | 69.104 |
| Vratsa | Abovground | 0.993 | 72.536 | 14.303 | 0.000 | 0.128 | 6.154 | 4.824 | 86.839 |
| Vratsa | Abovground | 1.049 | 70.955 | 15.115 | 0.000 | 0.135 | 6.503 | 5.098 | 86.070 |
| Bansko | Roots | 5.101 | 68.038 | 20.044 | 0.592 | 0.000 | 7.026 | 2.293 | 88.082 |
| Bansko | Roots | 5.391 | 66.902 | 21.182 | 0.626 | 0.000 | 7.426 | 2.424 | 88.084 |
| Vratsa | Roots | 4.730 | 60.930 | 6.457 | 3.940 | 14.724 | 11.977 | 18.655 | 67.387 |
| Vratsa | Roots | 4.998 | 59.391 | 6.824 | 4.163 | 15.560 | 12.696 | 19.714 | 66.215 |

**Table S2** Data used for the 2019 experiment.

| Location | Plantpart | 3-Methylbutanoic acid ( isovaleric acid) | 3-Methylpentanoic acid (3-methylvaleric acid | Germacrene D | n-Tetradecanal | Geranyl valerate | (2Z,6E)-Farnesyl acetate | Geranyl linalool | (9E,12E,15E)-Octadecatrien-1-ol | n-Octadecanol | (9E,12E,15E)-Octadecatrienal | 3,7,11,15-Tetramethyl-2-hexadecen-1-ol | (Z,Z)-9,12-octadecadienoic acid | (Z,Z,Z)-9,12, 15-octadecatrienoic acid | Monoterpenes  (monoterpenoids) | Sesquiterpenes (sesqoiterpenoid | Long-chain alkane | Fatty acid (esters, long-chain, methyl-branched | Others (esters, alcohol, fatty aldehyde) |
| --- | --- | --- | --- | --- | --- | --- | --- | --- | --- | --- | --- | --- | --- | --- | --- | --- | --- | --- | --- |
| Bansko | Stem | 0.544 | 0.205 | 3.283 | 18.352 | 1.023 | 2.338 | 3.591 | 24.085 | 3.388 | 5.322 | 4.567 | 3.735 | 5.378 | 1.095 | 10.813 | 10.238 | 11.630 | 64.468 |
| Bansko | Stem | 0.569 | 0.214 | 3.433 | 19.190 | 1.070 | 2.445 | 3.755 | 23.619 | 3.543 | 5.565 | 3.776 | 3.906 | 5.624 | 1.146 | 11.307 | 8.707 | 12.162 | 64.848 |
| Bansko | Root | 2.745 | 0.690 | 2.608 | 16.065 | 3.453 | 3.062 | 4.531 | 9.044 | 1.763 | 10.905 | 7.896 | 4.021 | 3.759 | 3.496 | 15.793 | 3.401 | 14.571 | 60.883 |
| Bansko | Root | 2.870 | 0.722 | 2.273 | 15.799 | 3.211 | 3.202 | 4.238 | 9.457 | 1.844 | 11.403 | 7.526 | 4.205 | 3.493 | 3.356 | 15.760 | 3.556 | 14.800 | 61.014 |
| Vratsa | Stem | 4.223 | 22.366 | 0.320 | 10.213 | 2.888 | 8.065 | 1.486 | 2.742 | 1.529 | 20.096 | 2.045 | 8.034 | 0.461 | 4.928 | 3.962 | 1.305 | 36.805 | 51.224 |
| Vratsa | Stem | 4.416 | 21.739 | 0.337 | 9.708 | 3.019 | 7.933 | 1.554 | 2.867 | 1.599 | 20.301 | 2.138 | 8.201 | 0.482 | 5.153 | 4.151 | 1.353 | 36.638 | 51.378 |
| Vratsa | Root | 28.305 | 6.807 | 0.722 | 12.065 | 2.054 | 3.710 | 6.009 | 5.852 | 5.021 | 7.097 | 1.066 | 0.954 | 1.065 | 3.072 | 6.901 | 2.723 | 40.167 | 45.559 |
| Vratsa | Root | 27.800 | 6.581 | 0.755 | 12.616 | 2.148 | 3.288 | 6.283 | 5.712 | 5.250 | 6.842 | 1.115 | 0.998 | 1.114 | 3.212 | 6.870 | 2.847 | 39.668 | 46.062 |

**Table S3** Data used for the hexane-extracted samples.

| Species | Location | Plantpart | 3-Methylbutanoic acid (isovaleric acid) | 3-Methylpentanoic acid (3-methylvaleric acid) | (2E)-Tridecen-1-al | n-tetradecanal | (2E,6E)-farnesyl acetate | n-hexadecoic acid | (Z,Z)-9,12-octadecadienoic acid | n- tetracosane | others (aldechide, alchohol, ethers) | Monoterpenoid | Sesquiterpenoids | fatty acid | Long-chain alkane |
| --- | --- | --- | --- | --- | --- | --- | --- | --- | --- | --- | --- | --- | --- | --- | --- |
| C. kellereri | Vratsa | leaves | 3.81 | 1.20 | 4.26 | 41.71 | 7.29 | 4.79 | 20.15 | 4.61 | 46.43 | 7.29 | 1.00 | 37.23 | 6.52 |
| C. kellereri | Vratsa | stems | 2.37 | 0.60 | 3.14 | 35.96 | 2.01 | 5.09 | 17.94 | 25.71 | 40.46 | 2.01 | 0.09 | 28.29 | 27.46 |
| C. kellereri | Vratsa | roots | 65.86 | 10.70 | 1.47 | 0.60 | 0.20 | 6.40 | 0.44 | * | 2.67 | 2.23 | 5.00 | 85.36 | 0.00 |
| C. kellereri | Bansko | leaves | 3.28 | 1.41 | 2.45 | 33.84 | 2.33 | 4.99 | 42.13 | 3.61 | 36.42 | 2.33 | 0.81 | 52.28 | 6.18 |
| C. kellereri | Bansko | stems | 7.83 | 0.34 | 1.49 | 50.95 | 2.04 | 3.60 | 24.13 | 3.18 | 52.98 | 2.04 | 0.74 | 36.63 | 5.15 |
| C. kellereri | Bansko | roots | 31.00 | 8.18 | 2.20 | 2.08 | 0.41 | 4.57 | 0.71 | * | 5.26 | 28.68 | 17.00 | 47.27 | 0.00 |
| C. ruber | Kazanluk | roots | 33.78 | 5.95 | 0.94 | 6.14 | 0.50 | 5.29 | 0.86 | * | 8.06 | 3.57 | 37.55 | 49.56 | 0.00 |
| C. ruber | Kazanluk | leaves | 4.37 | 1.24 | 0.23 | 36.44 | 3.29 | 14.16 | 21.97 | 9.34 | 36.66 | 3.29 | 2.90 | 44.74 | 9.87 |
| C. ruber | Kazanluk | stems | 6.96 | 0.88 | 0.93 | 44.79 | 4.17 | 9.68 | 18.28 | 7.32 | 45.92 | 4.17 | 0.42 | 36.83 | 10.36 |
| C. ruber | Kazanluk | flowers | 2.04 | 0.55 | 0.26 | 0.22 | 2.06 | 1.86 | 13.77 | 10.50 | 0.48 | 2.36 | 0.38 | 44.17 | 49.99 |
