# Supplementary material for: Phytochemical and biological investigations on Centranthus kellereri (Stoj., Stef. & T. Georgiev) Stoj. & Stef. and C. ruber (L.) DC. and their potential as new medicinal and ornamental plants
Source: PLoS One. 2023 Nov 7;18(11):e0293877. doi: 10.1371/journal.pone.0293877 (PMC10629660; doi:10.1371/journal.pone.0293877)
Supplement: Attachment — Submitted filename: Review PONE-D-23-25651_reviewer.pdf [file pone.0293877.s002.pdf]

PLOS ONE

Phytochemical and biological investigations on Centranthus kellereri (Stoj., Stef. & T. Georgiev) Stoj. & Stef. and C. ruber (L.) DC. and their potential as new medicinal and ornamental plants

--Manuscript Draft--

Manuscript Number: PONE-D-23-25651

Article Type: Research Article

Full Title: Phytochemical and biological investigations on Centranthus kellereri (Stoj., Stef. & T. Georgiev) Stoj. & Stef. and C. ruber (L.) DC. and their potential as new medicinal and ornamental plants

Short Title: Phytochemical and biological investigations on Centranthus kellereri and C. ruber

Corresponding Author: Valtcho D. Zheljazkov, PhD
Oregon State University
Corvallis, Oregon UNITED STATES

Keywords: endemics; Centranthus; embryology; DNA; fruits; leaves; SEM; seed viability, antioxidant

Abstract: Centranthus kellereri is a Bulgarian endemic plant species, found only in two locations in the world: The Balkans Mountains (Stara Planina), above the town of Vratsa, and The Pirin Mountains, above the town of Bansko, Bulgaria. The hypothesis of this study was that the populations of C. kellereri may represent genetically, phytochemically, and morphologically distinct forms and these will differentiate from Centranthus ruber. Furthermore, C. kellereri possibly imperfect embryology may preclude its more widespread distribution under natural conditions. This study revealed the phytochemical profile, antioxidant activity, embryology, surface microstructural morphological traits, and genetic differences between the C. kellereri plants from the only two natural populations and compares them to the ones of the related and better-known plant Centranthus ruber. The essential oil (EO) content in aboveground plant parts and in roots was generally low and the EO composition varied significantly as a function of plant part, year of sampling, location, and species. Methylvaleric acid was a major EO constituent in the C. kellereri EO, ranging between 60.2% and 71.7% of the total EO. The EO included monoterpenes, sequiterpenes, long-chain alkanes and fatty acids. Phytochemical analyses of plant tissue revealed the occurrence of 32 compounds that were tentatively identified as 6 simple phenolics, 18 flavonoids, 1 quinone, 1 lipid, 1 alkaloid, 2 diterpenes, and 3 triterpenes. There were differences in detected compounds between the C. kellereri plants at the two locations and between the roots and shoots in both species. The total phenols and flavonoids varied between the two species but were also dissimilar between the plants from the two populations of C. kelleri. Free radical scavenging activity, measured with ABTS and DPPH in aqueous and methanol extracts, had similar values; however, overall, C. kellereri from Vratsa showed the highest antioxidant activity while C. ruber had the lowest activity. Genetic analyses showed a clear differentiation between C. kellereri and C. ruber, and between the two populations of C. kellereri. Embryological studies revealed the peculiarities of the male and female generative spheres of the two species that were defined as being sexually reproducing. The pollen had high viability, however, the insufficient to low viability of seeds demonstrated possible high sensibility of C. kellereri to the environmental conditions, perhaps the main factor modifying and restricting the population sizes. The SEM analyses exposed differences in surface microstructural traits between the species (C. kellereri and C. ruber) but also between the two populations of C. kellereri. The observed dissimilarities in genetic makeup, micromorphological characteristics, and phytochemical composition strongly indicate that the two populations can be classified as distinct subspecies or varieties of C. kellereri: var. Pirinensis and var. Balkanensis. Further research is needed to introduce C. kellereri into culture and develop it as a high-value specialty crop or ornamental in order to conserve C. kellereri natural populations. Centranthus kellereri may be utilized as a source for phytochemicals of interest and as an ornamental plant, the same as C. ruber, however, possibly with a greater environmental plasticity and adaptation as evident by its current locations.

Powered by Editorial Manager® and ProduXion Manager® from Aries Systems Corporation

Order of Authors:	Valtcho D. Zheljazkov, PhD
	Ivanka B. Semerdjieva
	Daniela Borisova
	Elina Yankova-Tsvetkova
	Lyubka H. Koleva-Valkova
	Galya Petrova
	Ivayla Dincheva
	Fred Stevens
	Wenbin Wu
	Tess Astatkie
	Tanya Ivanova
	Albena Stoyanova
	Anatoli Dzhurmanski
Additional Information:	
Question	Response
**Financial Disclosure**<br><br>Enter a financial disclosure statement that describes the sources of funding for the work included in this submission. Review the submission guidelines for detailed requirements. View published research articles from *PLOS ONE* for specific examples.<br><br>This statement is required for submission and **will appear in the published article** if the submission is accepted. Please make sure it is accurate.	The author(s) received no specific funding for this work.

Powered by Editorial Manager® and ProduXion Manager® from Aries Systems Corporation

**Unfunded studies**<br>Enter: *The author(s) received no specific funding for this work.*<br><br>**Funded studies**<br>Enter a statement with the following details:<br>• Initials of the authors who received each award<br>• Grant numbers awarded to each author<br>• The full name of each funder<br>• URL of each funder website<br>• Did the sponsors or funders play any role in the study design, data collection and analysis, decision to publish, or preparation of the manuscript?<br>• **NO** - Include this sentence at the end of your statement: *The funders had no role in study design, data collection and analysis, decision to publish, or preparation of the manuscript.*<br>• **YES** - Specify the role(s) played.<br><br>* typeset	
**Competing Interests**<br><br>Use the instructions below to enter a competing interest statement for this submission. On behalf of all authors, disclose any competing interests that could be perceived to bias this work—acknowledging all financial support and any other relevant financial or non-financial competing interests.<br><br>This statement is required for submission and **will appear in the published article** if the submission is accepted. Please make sure it is accurate and that any funding sources listed in your Funding Information later in the submission form are also declared in your Financial Disclosure statement.<br><br>View published research articles from *PLOS ONE* for specific examples.	The authors have declared that no competing interests exist.

**NO authors have competing interests**<br><br>Enter: *The authors have declared that no competing interests exist.*<br><br>**Authors with competing interests**<br><br>Enter competing interest details beginning with this statement:<br><br>*I have read the journal's policy and the authors of this manuscript have the following competing interests: [insert competing interests here]*<br><br>* typeset	
**Ethics Statement**<br><br>Enter an ethics statement for this submission. This statement is required if the study involved:<br><br>• Human participants<br>• Human specimens or tissue<br>• Vertebrate animals or cephalopods<br>• Vertebrate embryos or tissues<br>• Field research<br><br>Write "N/A" if the submission does not require an ethics statement.<br><br>General guidance is provided below. Consult the submission guidelines for detailed instructions. **Make sure that all information entered here is included in the Methods section of the manuscript.**	N/A

Powered by Editorial Manager® and ProduXion Manager® from Aries Systems Corporation

**Format for specific study types**<br><br>**Human Subject Research (involving human participants and/or tissue)**<br>• Give the name of the institutional review board or ethics committee that approved the study<br>• Include the approval number and/or a statement indicating approval of this research<br>• Indicate the form of consent obtained (written/oral) or the reason that consent was not obtained (e.g. the data were analyzed anonymously)<br><br>**Animal Research (involving vertebrate animals, embryos or tissues)**<br>• Provide the name of the Institutional Animal Care and Use Committee (IACUC) or other relevant ethics board that reviewed the study protocol, and indicate whether they approved this research or granted a formal waiver of ethical approval<br>• Include an approval number if one was obtained<br>• If the study involved *non-human primates*, add *additional details* about animal welfare and steps taken to ameliorate suffering<br>• If anesthesia, euthanasia, or any kind of animal sacrifice is part of the study, include briefly which substances and/or methods were applied<br><br>**Field Research**<br><br>Include the following details if this study involves the collection of plant, animal, or other materials from a natural setting:<br>• Field permit number<br>• Name of the institution or relevant body that granted permission	
**Data Availability**<br><br>Authors are required to make all data underlying the findings described fully available, without restriction, and from the time of publication. PLOS allows rare exceptions to address legal and ethical concerns. See the PLOS Data Policy and FAQ for detailed information.	Yes - all data are fully available without restriction

A Data Availability Statement describing where the data can be found is required at submission. Your answers to this question constitute the Data Availability Statement and **will be published in the article**, if accepted.<br><br>**Important:** Stating 'data available on request from the author' is not sufficient. If your data are only available upon request, select 'No' for the first question and explain your exceptional situation in the text box.<br><br>Do the authors confirm that all data underlying the findings described in their manuscript are fully available without restriction?	
**Describe where the data may be found in full sentences. If you are copying our sample text, replace any instances of XXX with the appropriate details.**<br><br>• If the data are **held or will be held in a public repository**, include URLs, accession numbers or DOIs. If this information will only be available after acceptance, indicate this by ticking the box below. For example: *All XXX files are available from the XXX database (accession number(s) XXX, XXX.*).<br>• If the data are all contained **within the manuscript and/or Supporting Information files**, enter the following: *All relevant data are within the manuscript and its Supporting Information files.*<br>• If neither of these applies but you are able to provide **details of access elsewhere**, with or without limitations, please do so. For example:<br><br>*Data cannot be shared publicly because of [XXX]. Data are available from the XXX Institutional Data Access / Ethics Committee (contact via XXX) for researchers who meet the criteria for access to confidential data.*<br><br>*The data underlying the results presented in the study are available from (include the name of the third party*	Data will be available from Dr. Valtcho D. Zheljazkov, the corresponding author, after the manuscript has been published.

and contact information or URL).<br>• This text is appropriate if the data are owned by a third party and authors do not have permission to share the data.<br><br>* typeset	
Additional data availability information:	Tick here if the URLs/accession numbers/DOIs will be available only after acceptance of the manuscript for publication so that we can ensure their inclusion before publication.

Powered by Editorial Manager® and ProduXion Manager® from Aries Systems Corporation

Manuscript Click here to access/download;Manuscript;Manuscript.docx

Phytochemical and biological investigations on *Centranthus kellereri* (Stoj., Stef. & T. Georgiev) Stoj. & Stef. and *C. ruber* (L.) DC. and their potential as new medicinal and ornamental plants

Valtcho D. Zheljazkov[1*], Ivanka B. Semerdjieva[2,3], Daniela Borisova[4], Elina Yankova-Tsvetkova[3], Lyubka H. Koleva-Valkova[5], Galya Petrova[3], Ivayla Dincheva[6], Fred Stevens[7], Wenbin Wu[7], Tess Astatkie[8], Tanya Ivanova[9], Albena Stoyanova[10], Anatoli Dzhurmanski[11]

[1] Department of Crop and Soil Science, Oregon State University, Corvallis, OR 97331, USA.

[2] Department of Botany and Agrometeorology, Faculty of Agronomy, Agricultural University, 4000 Plovdiv, Bulgaria.

[3] Department of Plant and Fungal Diversity, Division of Flora and Vegetation, Institute of Biodiversity and Ecosystem Research, BAS, 23, Acad. G. Bonchev St., 1113 Sofia, Bulgaria.

[4] Administration of Vrachanski Balkan Nature Park, Executive Forest Agency, Ministry of Agriculture, Food and Forestry, 3000 Vratsa, Bulgaria.

[5] Department of Plant Physiology, Biochemistry and Genetics, Agricultural University, Mendeleev 12, 4000 Plovdiv, Bulgaria.

[6] Department of Agrobiotechnologies, Agrobioinstitute, Agricultural Academy, 8 "Dragan Tsankov" Blvd., 1164 Sofia, Bulgaria.

[7] Department of Pharmaceutical Sciences and the Linus Pauling Institute, Linus Pauling Science Center 435, Oregon State University, SW Campus Way, Corvallis, OR 97331, USA.

[8] Faculty of Agriculture, Dalhousie University, Truro, NS B2N 5E3, Canada.

[9] Department of Technology of Fats, Essential Oils, Perfumery and Cosmetics, University of Food Technologies, 26 Maritza, 4002 Plovdiv, Bulgaria.
[10] Department of Tobacco, Sugar, Vegetable and Essential Oils, Perfumery and Cosmetics, University of Food Technologies, 26 Maritza, 4002 Plovdiv, Bulgaria.
[11] Institute of Roses, Aromatic and Medicinal Plants, 6100 Kazanlak, Bulgaria
* Corresponding author: Valtcho.Jeliazkov@oregonstate.edu (Valtcho D. Zheljazkov)
Conflict of interest: Authors declare no conflict of interest.

These authors contributed equally to this work.

Abstract

Centranthus kellereri is a Bulgarian endemic plant species, found only in two locations in the world: The Balkans Mountains (Stara Planina), above the town of Vratsa, and The Pirin Mountains, above the town of Bansko, Bulgaria. Being endemic and endangered species precluded any significant research on it.

The hypothesis of this study was that the populations of *C. kellereri* may represent genetically, phytochemically, and morphologically distinct forms and these will differentiate from *Centranthus ruber*. Furthermore, *C. kellereri* possibly imperfect embryology may preclude its more widespread distribution under natural conditions. This study revealed the phytochemical profile, antioxidant activity, embryology, surface microstructural morphological traits, and genetic differences between the *C. kellereri* plants from the only two natural populations and compares them to the ones of the related and better-known plant *Centranthus ruber*.

Results: The essential oil (EO) content in aboveground plant parts and in roots was generally low and the EO composition varied significantly as a function of plant part, year of sampling, location, and species. Methylvaleric acid was a major EO constituent in the *C. kellereri* EO, ranging between 60.2% and 71.7% of the total EO.

The EO included monoterpenes, sequiterpenes, long-chain alkanes and fatty acids. Phytochemical analyses of plant tissue revealed the occurrence of 32 compounds that were tentatively identified as 6 simple phenolics, 18 flavonoids, 1 quinone, 1 lipid, 1 alkaloid, 2 diterpenes, and 3 triterpenes. There were differences in detected compounds between the *C. kellereri* plants at the two locations and between the roots and shoots in both species. The total phenols and flavonoids varied between the two species but were also dissimilar between the plants from the two populations of *C. kelleri*. Free radical scavenging activity, measured with ABTS and DPPH in aqueous and methanol extracts, had similar values; however, overall, *C. kellereri* from Vratsa showed the highest antioxidant activity while *C. ruber* had the lowest activity.

Genetic analyses showed a clear differentiation between *C. kellereri* and *C. ruber*, and between the two populations of *C. kellereri*. Embryological studies revealed the peculiarities of the male and female generative spheres of the two species that were defined as being sexually reproducing. The pollen had high viability, however, the insufficient to low viability of seeds demonstrated possible high sensibility of *C. kellereri* to the environmental conditions, perhaps the main factor modifying and restricting the population sizes. The SEM analyses exposed differences in surface microstructural traits between the species (C. *kellereri* and C. *ruber*) but also between the two populations of *C. kellereri*. The observed dissimilarities in genetic makeup, micromorphological characteristics, and phytochemical composition strongly indicate that the two populations can be classified as distinct subspecies or varieties of *C. kellereri*; var. *Pirinensis* and var. *Balkanensis*. Further research is needed to introduce *C. kellereri* into culture and develop it as a high-value specialty crop or ornamental in order to conserve *C. kellereri* natural populations. *Centranthus kellereri* may be utilized as a source for phytochemicals of interest and as an ornamental plant, the

same as *C. ruber*, however, possibly with a greater environmental plasticity and adaptation as evident by its current locations.

Keywords: endemics; Centranthus; embryology; DNA; fruits; leaves; SEM; seed viability

1. Introduction

Centranthus kellereri (Stoj. et Stef.) is an endemic plant species found exclusively at two locations in the world. The first location is in the Balkan Mountains (Stara Planina), situated between the village of Chelopek and the village of Pavolche above the town of Vratsa, Bulgaria [1]. The second location is in the Pirin Mountains, in the areas of Banski Suhodol, the Caves, and Academica locations, located above the town of Bansko, Bulgaria [2].

The two habitats of *C. kellereri* are characterized by plants spreading over mobile sandstone, rocky screes, with a northwestern exposure and an altitude ranging from 700 in the Balkan Mountains to 1,800 m in the Pirin Mountains. The species is a calcicole, with distinct mesoxerophytic features and is considered a pioneer in the vegetation cover of mesoxerophytic grassland formations. According to the EUNIS classification [3], the habitat of the species is H2.4 Mountainous sandstone and ultrabasic screes in the temperate zone. According to the Habitat Directive, it corresponds to natural habitat 8120 Sandstone screes and screes of sandstone shales from montane to alpine levels (Thlaspietea rotundifolii) [4].

This species is protected and included in Appendix 3 of the Law of Biological Diversity, as well as in the new edition of the Red Book of Bulgaria, Volume I. Plants and fungi, with the category of Critically Endangered [CR B2ab(v)]. Due to its status as an endemic and endangered species, there has been very little research conducted on *C. kellereri*. Consequently, there are almost no publications on its phytochemistry,

including the essential oil (EO), phytochemistry, micromorphological traits, genetics, embryology, or bioactivity. Furthermore, the intriguing phenomenon of *C. kellereri*'s restricted distribution to only two locations in two separate mountains, with an air distance of approximately 200 km between them, remains largely unexplained. Understanding the factors influencing this unique distribution pattern could potentially be linked to the species' embryology and reproductive strategies. Unfortunately, data on the features of the structures and running of the processes in the male and female generative spheres in the species of the *Centranthus* genus are limited and partial [5], and *C. kellereri* embryology has not been studied.

The closely related species *Centranthus ruber* DC has been extensively studied, with several reports on its bioactivity and the potential health benefits of its constituents [6, 7, 8, 9, 10]. However, not much is known about its phytochemistry. *Centranthus ruber* is a well-known and highly valued attractive ornamental plant that has a wide geographic and environmental range of growth, with wide adaptability, and has even triggered concerns regarding its invasive potential [11], [12]. Both *Centranthus kellereri* and *C. ruber* are perennial herbaceous plants with glabrous stems that can reach up to 50-120 cm in height. The stems are simple or branched in the upper half and are upright or ascending. The seeds of both species are one-seeded with a kite of feathery bristles [13].

Key features that distinguish *C. kellereri* from closely related species are the height of the plants, which can reach up to 120 cm. Since the species forms multiple equal stems from its base, the overall appearance of a single plant is like a tuft. The stems and leaves are bare and gray-green. The flowers are purplish-red to pink with tubular corollas ending in a linear spur. The corolla, together with the spur, is 2-2.5 cm long [13]. In Bulgaria, there are no closely related species, but in the global flora,

C. kellereri is sometimes described as a subspecies of *Centranthus longiflorus* Steven, specifically *Centranthus longiflorus* subsp. *kellereri* (Stoj., Stef. & Georgiev) I. Richardson (APG 2, APG3). According to one rare palynomorphological investigation of *C. kellereri*, *C. ruber*, and *C. longiflorus*, it becomes clear that pollen grains of *C. kellereri* have a larger size, narrower colpi, and thicker exine than those of *C. ruber* and *C. longiflorus* [14]. The results of the present study support the recognition of *C. kellereri* as a separate species rather than a subspecies of *C. longiflorus* [14]. Indeed, according to the official flora of Bulgaria, *C. kellereri* is a Bulgarian endemic species.

Natural products have proven to be a significant source of new compounds utilized in new drug development; however, their full potential has yet to be realized [15]. In a recent study, the aforementioned authors reviewed new drug development from 1981-2019, categorized as Biological, Natural Products, Natural Botanical Products, Semi-Synthetic Products, Synthetic Products, and Vaccines, all based on natural products [15].

Endemic and protected plant species are often inadequately studied and underutilized as sources for natural products. If a natural product of interest is identified in an endemic species, it can be introduced into culture, and agronomic management practices can be developed for its commercial production. Such an approach may provide several tangible benefits: (1) provides new sources for natural products of interest; (2) can serve as a source of income for human populations living in marginal lands, and (3) aids in the conservation of their often very limited populations.

Positive examples of this approach can be seen with Edelweiss (*Leontopodium alpinum* Cass.), a protected and rare species in European mountains, and *Leontopodium nivale*, which is endemic to the Pirin Mountains in Bulgaria, the

Prokletije Mountains in Montenegro, and the Central Apennines in Italy, and is the national flower of Switzerland. The medicinal species was domesticated and is being grown as a high-value crop in Switzerland for the production of extracts used in various consumer specialty products such as candy, drinks, and milk [16,17].

Indeed, a previous study showed that *Centranthus longiflorus*, a closely related species to *C. kellereri*, possesses sedative and anticonvulsant effects similar to those exhibited by diazepam [18]. Thus, the study of endemic and protected plant species can provide new sources of valuable natural products while aiding in their conservation.

The hypothesis of this study is that populations of *C. kellereri* may represent genetically, phytochemically, and morphologically distinct forms or varieties. These individuals may be genetically and chemically different from the well-known *Centranthus ruber*. Furthermore, *C. kellereri* possibly imperfect embryology may preclude its more widespread distribution under natural conditions. *Centranthus kellereri* could serve as a desirable source for phytochemicals of interest. Moreover, due to its taxonomic and morphological similarity to *C. ruber*, *C. kellereri* may have ornamental value. It grows and thrives under extreme conditions, such as on rock screes, and may therefore have potential as an ornamental plant in a wide range of environments compared with that of *C. ruber*.

The objectives of this study were to reveal: (1) the phytochemical and genetic differences of *C. kellereri* individuals from the two locations and compare them to the ones of *C. ruber*; (2) explore microstructural differences between the *C. kellereri* plants from the two populations using Scanning Electron Microscopy (SEM); (3) reveal the embryological characteristics of the species from the two populations as a possible explanation for the species limited distribution and endemicity.

2. Materials and methods

2.1. Plants material

The plant materials for this study were *Centranthus kellereri* sampled from the only two natural populations: (1) Banski Suhodol located in the northern part of Pirin Mountains (41°47´56.4´´N, 23°24´54.1´´E, 2017masl), above the town of Bansko, and (2) ZM “Vegdata” located in the Vrachanski Balkan Nature Park (43°08´22.67´´N, 23°35´59.74´´, 730masl of Stara Planina, (the Balkan Mountains), above the town of Vratsa.

As noted, *Centranthus kellereri* is a critically endangered and protected Bulgarian endemic species under the Biodiversity Act. Therefore, the research team obtained a special permit from the Ministry of Environment and Water (MEW) numbered 736/12.032018 issued to Dr. V.D. Jeliazkov (Zheljazkov), Mrs. Daniela Borisova, and Dr. C. Radoukova. *Centranthus ruber* was grown *ex-situ* and collected from the experimental field of the Institute of Roses, Essential Oil and Medical Crops, in the town of Kazanlak, Bulgaria (42°38´0.05´´N, 25°23´0.17´´E, 395masl). Representative subsamples were taken from *C. kellereri* individuals from the two populations and *C. ruber* specimens from the Institute of Roses, Essential Oil and Medical Crops, Kazanlak for DNA, phytochemical, metabolomic, antioxidant, anatomical (SEM), and embryological studies.

Plants were sampled for DNA, phytochemical, and some embryological analyses in May-June and August-September during two subsequent years. The samples for EO analyses were collected over two growing seasons, representing two different experiments. For DNA analyses, only plant leaves were collected, while root and rhizome plants were collected for phytochemical and antioxidant testing. For the embryology analyses, flower buds and open flowers were collected in June, and seeds

were collected in August-September when they reached maturity. Herbarium specimens of the *Centranthus kellereri* from the two natural populations and *Centranthus ruber* from Institute of Roses, Essential Oil and Medical Crops, in Kazanlak were deposited in the herbarium of the Agricultural University, Plovdiv, Bulgaria (herbarium numbers of the deposited Centranthus samples are 062637, 062638, 0622578, and 062636). Furthermore, some limited samples and extracts are available from the authors upon request.

2.2. Methods

2.2.1. Preparation of Centranthus kellereri and Centranthus ruber samples for the extraction of the essential oil (EO).

The samples of the two species were collected in June and were placed in a laboratory at a temperature of below 35°C for air-drying.

2.2.2 Essential Oil (EO) Extraction of Centranthus kellereri and Centranthus ruber Isolation of the essential oil (EO)

The EO was extracted via 3-h hydro distillation of 100 g *Centranthus* aboveground biomass or roots using Clevenger-type glass apparatus of the British Pharmacopoeia. The obtained EO was dried with anhydrous sodium sulfate, and placed in dark vials at 4°C until gas chromatographic (GC) analysis

2.2.3. Gas Chromatography Mass Spectrometry Flame Ionization Detection (GC-MS-FID) of essential oil (EO)

Oil samples in two replicates were studied by GC-FID and GC-MS as described earlier [19], with slight modifications. Briefly, GC analysis was performed on an Agilent 8890A system equipped with a FID and a DB-5 capillary column, 30 m × 0.25 mm, 0.25 μm, temperature programmed as follows: 40-300°C at 5°C/min. The carrier

gas was nitrogen at a flow of 1.0 ml/min; the injector port and the detector temperatures were 230°C and 280°C, respectively. Samples were injected by splitting (split ratio 30:1).

GC/MS analysis was performed [illegible] an Agilent 5977B 6890 /5972 system with a DB-5 capillary column (30 m × 0.25 mm; 0.25 µm), as the operating conditions were the same as described above. The carrier gas was He (flow 1.0 ml/min). Mass spectra were taken at 70 eV in electron impact (EI) mode and the scan mass range was from 40-400 m/z. The ionization source, the transfer line, and the injector temperatures were set at 230°C, 280°C, and 250°C, respectively.

The quantitative data were obtained from the electronic integration of the FID peak areas. The components of the oil samples were identified by their retention time, retention indices, relative to C8-C40 n-alkanes, matching with the Adams [20] and NIST'08 [21] libraries and by comparison of their mass spectra with data already available in the literature. The percentage of the identified compounds was computed from the GC peaks areas without any correction factors.

2.2.4. Phytochemical analyses

Dried above ground plant parts and roots were extracted and analyzed essentially as described by [22]. Extract constituents were characterized by retention time, accurate mass, isotopic pattern, and MS/MS spectra and identified by LC-MS/MS comparison with 500 authentic standards (Natural Products Library, Enzo Life Sciences). The distribution and relative abundance of the phytochemicals was visualized by the open-access software tool MetaboAnalyst 5.0 [23].

2.2.5. DNA extraction

DNA extraction

Total genomic DNA was isolated from silica-dried leaves using standard CTAB extraction procedure [24]. Quantitative and qualitative tests of the DNA were done using a Nanodrop ND-1000 spectrophotometer. DNA samples were diluted to 25ng/μL, and stored at -20°C before amplification.

ISSR amplification product analysis

Firstly, the polymorphism of 30 markers was tested and then seven polymorphic and reproducible ISSR primers (Microsynth, Balgach, Switzerland) were selected (Table 1). Polymerase chain reactions (PCR) were performed according to Petrova et al. (2014). PCR reactions were carried out in a Techne TC-5000 gradient thermal circler (Techne, Staffordshire, UK). The reproducibility of the technique was tested by replicating each amplification reaction twice. Amplification products were separated on 2% agarose gels stained by incorporating 1% GelRed (Biotium Inc., USA) at 1.5h, 135V along with 100 bp Plus DNA Ladder (Thermoscientific, Vilnius, Lithuania). The DNA fragments were visualized under UV light and further analyzed with a video image analyzer (BioImaging Systems, Cambridge, UK).

Data analysis

ISSR bands (clear and unambiguous) were scored using the binary system as present (1) versus absent (0). To learn about the genetic diversity among populations, we calculated the percentage of polymorphic loci (*P*), genetic diversity (*h*), unbiased genetic diversity and Shannon's information index (*SI*) in the software GenALEx 6.5 [25]. The partitioning of genetic variability among populations, and Hierarchical Analysis of Molecular Variance (AMOVA) was performed with GenAlEx 6.5 by resembling 999 times. Genetic differentiation among populations (F_{PT}) based on the Euclidean distances was calculated with GenAlEx 6.5 and the latter were used to carry out a Principal Coordinate Analysis (PCoA). To investigate the genetic structure and

the degree of admixture between each sample and between the three *Centranthus* populations, the Bayesian clustering method of STRUCTURE 2.3.4 [26] was used. The simulations were conducted with a burn-in of 500 000 generations followed by Markov Chain Monte Carlo (MCMC) of 1 000 000 iterations. The *K* value was set to run from 1 to 10, each with 10 replications for reliability. The optimal number of clusters was estimated using the [27] in Structure Harvester [28].

Table 1. ISSR primers used for analysis of genetic diversity of different *Centranthus* species in Bulgaria

Primer	Sequence 5'- 3'	Total number of bands	Number of polymorphic bands	Polymorphism (%)	Annealing temperature (ºC)
ISSR1	$(AG)_{10}C$	10	7	70	60
ISSR2	$(AG)_8YT$	9	9	100	60
ISSR3	$(AC)_8G$	10	8	80	55
ISSR4	$(AC)_8YG$	8	6	75	60
ISSR5	$(AC)_8YT$	12	10	83	60
ISSR6	$(AC)_8YG$	9	7	78	55
ISSR7	$(AC)_8YA$	11	7	64	60
Total		69	54	79	

2.2.6. Embryological analyses.

To estimate the reproductive capacity of the two target *Centranthus* species embryological studies (revealing the features of male and female gametophytes and the processes running in the reproductive structures) and an evaluation of basic parameters of the reproductive potential (pollen and seed viability) were carried out. For the purpose of the embryological study, material (flower buds and flowers at different stages of development), collected from the only two populations of *Centranthus kellereri* and a cultivated *C. ruber* described in the section 2.1. were fixed in FAA (formalin: glacial acetic acid: 70% ethanol in a ratio of 5:5:90 parts). Subsequent processing was carried out according to the classical paraffin methods [29]. Cuttings on the initial material with a thickness of 8 to 18 µm (depending on the

developmental stage) were made with a rotary microtome “Leica”. The staining was performed with hematoxylin [30]. Permanent slides were prepared by including the cuts in Enthelan. The observations on the permanent slides were studied using light microscope Olympus CX21 (Olympus Corporation, Tokyo, Japan). The photographs were made with “Infinity lite” digital camera, 1.4 Mpx.

Polen viability

For estimation of pollen viability, the mature pollen grains in 30 anthers of the populations of the two *Centranthus* species were counted (on visible field at magnification 100x or 400x depending on their size). The count was performed on the permanent slides described above: the pollen grains as viable were estimated the ones with a distinguishable vegetative and generative cell with clear sculpture, and as non-viable - the degenerate dark-colored, deformed pollen grains (i.e., sterile, non-functional).

Seed viability testing

A rapid 24-hour test known as the tetrazolium test [31] was used to evaluate seed viability. For this purpose, embryos isolated from mature seeds pre-soaked in water at 30-35°C were incubated in a diluted 1% solution of 2,3,5-triphenyltetrazolium chloride for 24 hours at 25°C. The initially colorless tetrazolium solution turned red upon contact with the hydrogen from the respiratory enzymes in the seeds (embryos). Embryos with active respiratory activity were colored in red by the tetrazolium solution and were considered viable. The more intensive the coloration, the more active was the respiratory activity of the embryo, while pink-colored embryos indicated lower viability than those colored in dark red. Colorless embryos were considered non-viable. The assessment was made according to the criteria for interpretation of tetrazolium test results [32], based on the color intensity and

localization of the colored and uncolored parts after testing: embryos stained in dark red and pink and partially stained embryos (only the root tip colored in red) were considered viable, while colorless embryos or partially stained ones (only the top of the embryo – cotyledons are colored in red) were considered non-viable. Observations and micrographs were taken using a light microscope Olympus CX21 and an "Infinity lite" digital camera with 1.4 Mpx.

2.2.7. Scanning electron microscopy (SEM) analysis

The scanning electron microscope (SEM) analysis was performed using an FEI Quanta 600 SEM at the Oregon State University Microscopy Facility. The samples for SEM analysis were prepared by immersing subsamples into a fixative containing 1% paraformaldehyde and 2.5% glutaraldehyde in 0.1M sodium cacodylate buffer with a pH of 7.4. The fixation procedure continued for 2 hours, followed by two rinses in 0.1M cacodylate buffer for 15 minutes each. Afterwards, the samples were subjected to a dehydration series in acetone (10%, 30%, 50%, 70%, 90%, 95%, and 100-100%), for 10-15 minutes each, followed by critical point drying (two ‘bomb flushes’ at chamber pressure to 5°C and filling the chamber with CO2). After dehydration, the samples were left to vent for 5 minutes, and the same procedure was repeated. The dry samples were then mounted onto an aluminum SEM stub with double-stick carbon tape. Samples on the sample holders were sputter-coated with a Cressington 108A sputter coater from Ted Pella with Au/Pd, 60/40 mix. The description of cells, shape, as well as the structure of the surfaces of leaves, stem, and seeds were determined according to [33].

2.2.8. Analysis of Total Phenols, Total Flavonoids and Antioxidant activity

The roots of three plant populations were studied; *Centranthus kellereri* collected from two natural populations, in the Pirin Mountains above the town of Bansko, and

in the Balkan Mountains above the town of Vratsa. In addition, *C. ruber* samples were collected from the Institute of Roses, Essential Oil and Medical Crops, in the town of Kazanlak.

Plant material and extraction method

One gram of ground and dried roots and 10 mL of solvent were used to extract phenolic and flavonoid compounds. Each variant was placed in a capped tube for the duration of the extraction. Three different solvents were used: 70% acidic methanol, 70% ethanol, and distilled water. Each variant was performed in 4 replicates. The ratio of dry plant material to extractant was 1:10 (1 g to 10 mL). The extraction lasted for 24 hours at room temperature and then for 6 days at 4°C in the refrigerator. After extraction, the samples were filtered, and the resulting filtrates were used for analysis.

Analysis of total phenols

Quantitative determination of total phenols was performed by the method of [34] with minor modifications. A 40 µL sample (filtrate prepared as described above), 3160 µL of distilled water, 200 µL of Folin-Ciocalteu reagent and 300 µL of 20% (w/v) $NaCO_3$ were mixed in a tube. The tubes were incubated for 30 minutes at 75°C and after cooling, the absorbance was recorded spectrophotometrically at 760 nm. The quantity of total phenols was calculated from a standard gallic acid curve with the formula $y=1.421x + 0.0074$ ($R^2=0.9997$) and was expressed as mg of gallic acid equivalents per granular dry weight.

Analysis of total flavonoids

Total flavonoids were determined using the method described by [35]. For this, 1 mL of sample (filtrate), 4 mL of distilled water, and 300 µL of 5% (v/v) NaNO2 were mixed. After 5 minutes, 300 µL of 10% AlCl3 was added, and after an additional 6 minutes, 2 mL of 1M NaOH was added. The volume was immediately made up to 10

mL with distilled water. The absorbance was measured spectrophotometrically at a wavelength of 510 nm. The total amount of flavonoids was calculated from a standard quercetin curve with the formula y=0.0776x + 0.0244 (R^2=0.9963) and was expressed as mg of quercetin equivalents per gram of dry weight.

DPPH assay

To prepare the DPPH reagent, 0.012 g was dissolved in 100 mL of absolute ethanol and placed in an ultrasonic bath two times for 15 min. This solution was used to determine the antiradical activity of plant extracts by the method of [36] with minor changes. For this purpose, 1.8 mL of absolute ethanol, 0.6 mL of DPPH solution, and 0.6 mL of sample were mixed in the reaction tubes. Absorption was recorded after 10 minutes at 517 nm against absolute alcohol. The DPPH radical scavenging activity of the sample was expressed as mg Trolox equivalent antioxidant capacity (TEAC) by formula obtained from standard curve (y = -0.1954x + 0.708 R^2 = 0.9858).

ABTS assay

The ABTS assay included the preparation of stock and working solutions and following an approved procedure. The method was a modification of [37]. A stock solution of ABTS (7 mM) and $K_2S_2O_8$ (140 mM) was first prepared. For this purpose, 96.04 mg of ABTS was dissolved in 25 mL of acetic acid buffer with pH 4.5. Potassium persulfate ($K_2S_2O_8$) 378.4 mg was dissolved in 10 mL acetic acid buffer with pH 4.5. Both stock solutions were kept in dark (0-4°C).

Preparation of ABTS working solution

Five milliliters of ABTS (7mM) and 88 µL of $K_2S_2O_8$ (140 mM) stock solution were mixed and kept at room temperature for 16 h in dark to make an ABTS reaction solution. Then ABTS reaction solution was diluted with distilled water on proportion 1:44 (v/v). The absorbance at 734 nm was 0.700±0.05.

ABTS assay procedure

A 100 mL sample (or Trolox standard) and 2.900 mL ABTS working solution were mixed in tubes. The tubes were incubated for 5 min and then the absorbance was measured at 734 nm wavelength. The Trolox was used as a standard and distilled water as the blank control. The ABTS radical scavenging activity of sample was expressed as mg Trolox equivalent antioxidant capacity (TEAC) by formula obtained from standard curve (y=-0.0446x + 0.8223 R^2 = 0.984).

2.2.9. Statistical Analyses of EO samples

Samples from the 2018 growth season experiment

(a) For *C. kellereri* species, the effects of (1) Location (population) (2 levels: Bansko and Vratsa), and (2) Plant part (2 levels: Aboveground and Roots) on 8 compounds (furfural, 3-methylbutanoic acid (isovaleric acid), 3-methylpentanoic acid (3-methylvaleric acid), benzene acetaldehyde (hyacinthin), borneol, others (aldechyde, alcohol, ethers), monoterpenoid, and methyl-branched fatty acid) was determined using a nested design where Plantpart is nested in Location. The components of the model are shown in the Source of Variation row of Table 2. The multiple means comparison results are shown in Table 3.

(b) For *C. ruber* species, where the data were collected only from Kazanlak location and from Roots Plantpart, only descriptive statistics (average of the two replications) are reported in Table 4.

Table 2. 2018 *Centranthus kellereri* species: ANOVA *p*-values that show the significance of the effects of Location and Plant part nested in Location on 8 compounds. Significant effects that require multiple means comparison are shown in bold.

Compound	Source of variation

	Location	Plant part (Location)
Furfural	0.001	0.001
3-Methylbutanoic acid (isovaleric acid)	0.308	0.001
3-Methylpentanoic acid (3-methylvaleric acid)	0.002	0.001
Benzene acetaldehyde (hyacinthin)	0.216	0.001
Borneol	0.256	0.001
Others (aldechyde, alcohol, ethers)	0.760	0.001
Monoterpenoid	0.008	0.001
Methyl-branched fatty acid	0.013	0.001

Table 3. 2018 growing season *C. kellereri* species: Mean concentration (%) of furfural, 3-methylbutanoic acid (isovaleric acid) (Methy-I), 3-methylpentanoic acid (3-methylvaleric acid) (Methy-3-M), Benzene acetaldehyde (hyacinthin) (Benzene), borneol, others (aldechyde, alcohol, ethers) (Other), monoterpenoid (Monoter), and methyl-branched fatty acid (Methyl-b) obtained from the four combinations of Location and Plant part.

Plant part (Location	Furfural	Methy-I	Methy-3-M	Benzene	Borneol	Other	Monoter	Methyl-b
Aboveground (Bansko)	4.9 a*	62.4c	6.22c	3.79a	14.33a	11.59 a	18.26a	68.67c
Aboveground (Vratsa)	1.02 b	71.7 a	14.7 b	0.00 c	0.13 b	6.33 c	4.96 b	86.45b
Roots (Bansko)	5.25 a	67.5 b	20.61 a	0.61 b	0.00 b	7.23 b	2.36 c	88.08a
Roots (Vratsa)	4.86 a	60.16 c	6.64 c	4.05 a	15.14 a	12.34 a	19.18 a	66.80 d

*Within each column, means sharing the same letter are not significantly different at the 5% level of significance.

Table 4. 2018 *Centranthus ruber*, Kazanlak location, and roots Plant part: Mean concentration (%) of the 8 compounds listed in the table.

Compound	Concentration (%)
Furfural	5.31
3-Methylbutanoic acid (isovaleric acid)	77.53
3-Methylpentanoic acid (3-methylvaleric acid)	10.08
Benzene acetaldehyde (hyacinthin)	0.58
Borneol	0.00
Others (aldechyde, alcohol, ethers)	7.22
Monoterpenoid	3.33
Methyl-branched fatty acid	87.61

Samples from the 2019 growing season experiment

In *Centranthus kellereri* species, the effects of (1) Location (population) (2 levels: Bansko, and Vratsa), and (2) Plant part (2 levels: Root and Stem) on 18 compounds (3-methylbutanoic acid (isovaleric acid), 3-methylpentanoic acid (3-methylvaleric acid), germacrene D, n-tetradecanal, geranyl valerate, (2Z,6E)-farnesyl acetate, (E,E)-geranyl linalool, (9E,12E,15E)-octadecatrien-1-ol, n-octadecanol, (9E,12E,15E)-octadecatrienal, 3,7,11,15-tetramethyl-2-hexadecen-1-ol, (Z,Z)-9,12-octadecadienoic acid, (Z,Z,Z)-9,12, 15-octadecatrienoic acid, monoterpenes, monoterpenoids, sesquiterpenes, sesqoiterpenoids, long-chain alkane, fatty acid (esters, long-chain, methyl-branched), and others (esters, alcohol, fatty aldehyde)) was determined using a nested design where Plant part is nested in Location. The components of the model are shown in the Source of Variation row of Table 5. The multiple means comparison results are shown in Tables 6 and 7.

The analysis was conducted using the GLM procedure [38]. For each response variable, the validity of model assumptions on the error terms was verified by examining the residuals as described [39]. When normal distribution assumption is violated, an appropriate transformation was applied; however, their means reported in the tables are back-transformed to the original scale. Since Plantpart nested in

Location effect was significant ($p < 0.05$) in all compounds, multiple means comparison was conducted using the Fisher's LSD method at the 5% level of significance to compare all combinations of location and plant part. Even if the effect of Location is significant, if the effect of Plant part(Location) is significant, multiple means comparison is done only Plant part (Locatrion) because the differences among the plant parts varies with location.

Centranthus ruber

Since there is no enough error degree of freedom to do a test of hypothesis to test if there is a significant difference between the Aboveground and Roots, only descriptive statistics (averages of the 2 replications) are shown below (Table 8).

Table 5. 2019 Experiment. *Centranthus kellereri* species: ANOVA *p*-values that show the significance of the effects of Location and Plant part nested in Location on 18 compounds. Significant effects that require multiple means comparison are shown in bold.

Compound	**Source of variation**	
	Location	Plant part (Location)
3-Methylbutanoic acid (isovaleric acid)	0.001	0.001
3-Methylpentanoic acid (3-methylvaleric acid)	0.001	0.001
Germacrene D	0.001	0.004
n-Tetradecanal	0.001	0.002
Geranyl valerate	0.001	0.001
(2Z,6E)-Farnesyl acetate	0.001	0.001
(E,E)-Geranyl linalool	0.149	0.001
(9E,12E,15E)-Octadecatrien-1-ol	0.001	0.001
n-Octadecanol	0.001	0.001
(9E,12E,15E)-Octadecatrienal	0.001	0.001
3,7,11,15-Tetramethyl-2-hexadecen-1-ol	0.001	0.001
(Z,Z)-9,12-octadecadienoic acid	0.002	0.001
(Z,Z,Z)-9,12, 15-octadecatrienoic acid	0.001	0.001

Monoterpenes (monoterpenoids)	0.001	0.001
Sesquiterpenes (sesqoiterpenoids)	0.001	0.001
Long-chain alkane	0.001	0.001
Fatty acid (esters, long-chain, methyl-branched)	0.001	0.001
Others (esters, alcohol, fatty aldehyde)	0.001	0.001

Table 6. 2019 Experiment. *Centranthus kellereri* species: Mean concentration (%) of 3-methylbutanoic acid (isovaleric acid) (3-Meth-Iso), 3-methylpentanoic acid (3-methylvaleric acid) (3-Meth-3-M), germacrene D (Ger-D), n-tetradecanal (n-Tet), geranyl valerate (Ger-val), (2Z,6E)-farnesyl acetate (2Z,6E), (E,E)-geranyl linalool (E,E), (9E,12E,15E)-octadecatrien-1-ol (Oct-1), and n-octadecanol (n-Oct) obtained from the four combinations of Location and Plant part.

Plantpart/ Location	3-Meth-Iso	3-Meth-3-M	Ger-D	n-Tet	Ger-val	2Z,6E	E,E	Oct-1	n-Oct
Root (Bansko)	2.81 c*	0.71 c	2.44b	15.93 b	3.33a	3.13b	4.38b	9.25b	1.80c
Root (Vratsa)	28.05a	6.69 b	0.74c	12.34c	2.10c	3.50b	6.15a	5.78c	5.14a
Stem (Bansko)	0.56 d	0.21 c	3.36a	18.77a	1.05 d	2.39 c	3.67c	23.85a	3.47 b
Stem (Vratsa)	4.32 b	22.05a	0.33d	9.96 d	2.95 b	8.00 a	1.52d	2.80 d	1.56c

*Within each column, means sharing the same letter are not significantly different at the 5% level of significance.

Table 7. 2019 Experiement. *Centranthus kellereri* species: Mean concentration (%) of (9E,12E,15E)-octadecatrienal (Octad), 3,7,11,15-tetramethyl-2-hexadecen-1-ol (3,7,11,15), (Z,Z)-9,12-octadecadienoic acid (Z,Z), (Z,Z,Z)-9,12, 15-octadecatrienoic acid (Z,Z,Z), monoterpenes (monoterpenoids) (Mono), sesquiterpenes (sesqoiterpenoids) (Sesq), long-chain alkane (Long), fatty acid (esters, long-chain,

methil-branched) (Fatty), and others (esters, alcohol, fatty aldehyde) (Others) obtained from the four combinations of Location and Plantpart.

Plant part (Location)	Octad	3,7,11,15	Z,Z	Z,Z,Z	Mono	Sesq	Long	Fatty	Others
Root (Bansko)	11.2 b	7.7 a	4.1b	3.6b	3.4 b	15.8 a	3.5b	14.7 c	60.9 b
Root (Vratsa)	6.97c	1.1 d	0.98 c	1.1 c	3.1 b	6.9c	2.8b c	39.9 a	45.8 d
Stem (Bansko)	5.44d	4.2 b	3.8b	5.5 a	1.12 c	11.1 b	9.5 a	11.9 d	64.7 a
Stem (Vratsa)	20.2a	2.1 c	8.12 a	0.5d	5.04 a	4.06 d	1.3c	36.7 b	51.3 c

Within each column, means sharing the same letter are not significantly different at the 5% level of significance.

Table 8. 2019 Experiment. *Centranthus ruber* species (Kazanlak): Mean concentration (%) of the compounds measured in the aboveground and roots.

Compound name	**Aboveground**	**Root**
3-Methylbutanoic acid (isovaleric acid)	0.50	8.68
3-Methylpentanoic acid (3-methylvaleric acid)	0.20	2.09
Germacrene D	4.01	0.48
n-Tetradecanal	20.5	6.04
Geranyl valerate	0.93	1.30
(2Z,6E)-Farnesyl acetate	3.15	7.68
(E,E)-Geranyl linalool	4.29	3.77
(9E,12E,15E)-Octadecatrien-1-ol	6.58	9.26
n-Octadecanol	3.08	4.71
(9E,12E,15E)-Octadecatrienal	22.51	18.73
3,7,11,15-Tetramethyl-2-hexadecen-1-ol	3.95	3.24
(Z,Z)-9,12-octadecadienoic acid	3.40	5.88

(Z,Z,Z)-9,12, 15-octadecatrienoic acid	4.90	6.06
Monoterpenes (monoterpenoids)	1.00	0.57
Sesquiterpenes (sesqoiterpenoids)	10.87	9.93
Long-chain alkane	8.47	4.89
Fatty acid (esters, long-chain, methil-branched)	10.52	25.25
Others (esters, alcohol, fatty aldehyde)	67.31	57.89
Total	98.16	98.52

Since only the *C. kellereri* species, Vratsa location and *C. ruber* and the two Plant parts (leaves and stems) have replications, it is not possible to do ANOVA. Instead, descriptive statistics (averages for these two Plant parts, and the actual observed values) are reported in Tables 9 and 10.

Table 9. Chemical profile of the hexane-extracted samples: Mean concentration (%) of 3-methylbutanoic acid (isovaleric acid) (3-Meth-Iso), 3-methylpentanoic acid (3-methylvaleric acid) (3-Meth-3-M), (2E)-tridecen-1-al (2E), n-tetradecanal (n-Tetrad), (2E,6E)-farnesyl acetate (2E,6E), n-hexadecoic acid (n-Hex), and (Z,Z)-9,12-octadecadienoic acid (Z,Z).

Species	Location	Plantpart	3-Meth-Iso	3-Meth-3-M	2E	n-Tetrad	2E,6E	n-Hex	Z,Z
C. kellereri	Vratsa	leaves	3.81	1.20	4.26	41.7	7.29	4.79	20.2
C. kellereri	Vratsa	stems	2.37	0.60	3.14	36.0	2.01	5.09	17.9
C. kellereri	Vratsa	roots	65.9	10.70	1.47	0.60	0.20	6.40	0.44
C. kellereri	Bansko	leaves	3.28	1.41	2.45	33.8	2.33	4.99	42.1
C. kellereri	Bansko	stems	7.83	0.34	1.49	51.0	2.04	3.60	24.1
C. kellereri	Bansko	roots	31.0	8.18	2.20	2.08	0.41	4.57	0.71
C. ruber	Kazanlak	roots	33.8	5.95	0.94	6.14	0.50	5.29	0.86
C. ruber	Kazanlak	leaves	4.37	1.24	0.23	36.4	3.29	14.2	22.0
C. ruber	Kazanlak	stems	6.96	0.88	0.93	44.8	4.17	9.68	18.3
C. ruber	Kazanlak	Flowers	2.04	0.55	0.26	0.22	2.06	1.86	13.8

Table 10. Chemical profile of the hexane-extracted samples: Mean concentration (%) of n-tetracosane (n-Tetrac), others (aldechyde, alchohol, ethers) (Other), monoterpenoid (Mono), sesquiterpenoids (Sesq), fatty acid (Fatty), and long-chain alkane (Long).

Species	Location	Plantpart	n-Tetrac	Other	Mono	Sesq	Fatty	Long
C. kellereri	Vratsa	Leaves	4.61	46.43	7.29	1.00	37.23	6.52
C. kellereri	Vratsa	Stems	25.71	40.46	2.01	0.09	28.29	27.46
C. kellereri	Vratsa	Roots	nd	2.67	2.23	5.00	85.36	0.00
C. kellereri	Bansko	Leaves	3.61	36.42	2.33	0.81	52.28	6.18
C. kellereri	Bansko	Stems	3.18	52.98	2.04	0.74	36.63	5.15
C. kellereri	Bansko	Roots	nd	5.26	28.68	17.00	47.27	0.00
C. ruber	Kazanlak	Roots	nd	8.06	3.57	37.55	49.56	0.00
C. ruber	Kazanlak	Leaves	9.34	36.66	3.29	2.90	44.74	9.87
C. ruber	Kazanlak	Stems	7.32	45.92	4.17	0.42	36.83	10.36
C. ruber	Kazanlak	Flowers	10.50	0.48	2.36	0.38	44.17	49.99

nd = not detected.

2.2.10. Antioxidant activity statistical analyses

A 3 x 3 factorial design with 4 replications was used to determine the main and interaction effects of Population (Kazanlak, Bansko, and Vratsa) and Extraction method (EtOH70%, MeOH70%+HCL, and Water) on flavonoids, polyphenols, and two antioxidant activities (ABTS and DPPH). The analyses were conducted using the Mixed Procedure of SAS (SAS Institute Inc. 2014). Since the interaction effect of Population and Extraction method was highly significant (p-value < 0.01) for all four response variables, multiple means comparison was done to compare the nine combinations of Population and Extraction method using the lsmeans statement at the 5% level of significance (Table 11 and 12). For all four response variables, normal distribution and constant variance assumptions on the error terms were verified by examining the residuals as described in [39].

Table 11. Mean flavonoids (mgQE/gDW), polyphenols (mgGAE/gDW), ABTS (mgTE/gDW), and DPPH (mgTE/gDW) obtained from the nine combinations of Population and Extraction Method. Within each column, means sharing the same letter are not significantly different.

Species	Population	Extraction method	Flavonoids	Polyphenols	ABTS	DPPH
C. ruber	Kazanlak	EtOH70%	226.2 b	7.976 a	1.808 a	0.107 c
C. ruber	Kazanlak	MeOH70%+HCL	81.3 e	0.585 d	0.667 d	0.132 bc
C. ruber	Kazanlak	Water	128.7 d	3.229 c	1.816 a	0.149 bc
C. kellererii	Bansko	EtOH70%	220.7 b	6.382 b	1.777 a	0.227 a
C. kellereri	Bansko	MeOH70%+HCL	88.8 e	0.771 d	0.881 c	0.154 b
C. kellereri	Bansko	Water	155.2 c	3.637 c	1.814 a	0.235 a
C. kellereri	Vratsa	EtOH70%	302.9 a	6.331 b	1.757 a	0.264 a
C. kellereri	Vratsa	MeOH70%+HCL	156.2 c	0.829 d	1.090 b	0.253 a
C. kellereri	Vratsa	Water	208.6 b	3.699 c	1.812 a	0.232 a

Table 12. *p*-values of the ANOVA that show the significance of the main and interaction effects of population and extraction method.

Source of variation	Flavonoids	Polyphenols	ABTS	DPPH
Population	0.001	0.028	0.001	0.001
Extraction Method	0.001	0.001	0.001	0.005
Population*Extraction Method	0.006	0.001	0.001	0.001

3. RESULTS

3.1. Population genetic diversity study

Population genetic diversity

The 7 ISSR primers used generated 69 polymorphic bands, ranging in size between 150 and 3000 bp (Table 1). The primer $(AC)_8YT$ produced the highest number of bands (12) whereas the primer $(AC)_8YG$ produced the lowest number of bands (8), (Table 1). The percentage of polymorphic loci (*P*) varied from 66.67% (*Centranthus kellereri*, Bansko) to 75.00% (*Centranthus kellereri*, Vratsa). The *Centranthus kellereri* Vratsa population exhibited the highest values of genetic indexes h, h_u and SI of 0.255, 0.261 and 0.383, respectively (Table 13).

Population genetic structure and differentiation

Principal coordinates analysis (PCoA) was carried out to provide spatial representation of the genetic structure of the three *Centranthus* populations. The first two principal coordinates accounted for 46.93% of the total variance (28.76% and 18.17%, respectively), Fig. 1. The results showed a clear differentiation between species; populations of different *Centranthus* localities were genetically distant and the individuals form three major groups, only few *Centranthus kellereri* individuals fell into the *Centranthus ruber* population cluster. Population genetic structure was further studied using a Bayesian clustering algorithm; perform in STRUCTURE (Fig.

2). According to the method described by Evanno et al. (2005), the investigated *Centranthus* individuals were optimally divided into three main clusters (Fig. 2A, B). The result of AMOVA showed that 66% of the variation to reside within populations, and 34% among investigated populations. A significant genetic difference was found among investigated populations ($F_{PT} = 0.341$, $P < 0.001$) (Table 14).

Table 13. Genetic diversity indices of investigated *Centranthus* populations

Population	*P* (%)	*h*	h_u	SI	Private bands
Centranthus kellereri, Bansko	66.67	0.175 (0.037)	0.179 (0.038)	0.275 (0.053)	0
Centranthus kellereri, Vratsa	75.00	0.255 (0.040)	0.261 (0.041)	0.383 (0.055)	3
Centranthus ruber	70.83	0.238 (0.039)	0.244 (0.040)	0.360 (0.055)	3
Mean (SE)	70.83 (2.41)	0.222 (0.022)	0.228 (0.023)	0.339 (0.031)	2

P: percentage of polymorphic loci; *h*: genetic diversity, h_u: unbiased genetic diversity; SI: Shannon's information index.

Table 14. Analysis of molecular variance based on the ISSR markers for the three populations of *Centranthus*

Source of variance	df	*SS*	MS	Variance component	Percentage total (%)	*F* statistic	*P*
Among populations	2	66.500	33.250	1.516	34	$F_{PT} = 0.341$	0.001
Within populations	57	166.900	2.928	2.928	66		
Total	59	233.400		4.444	100		

Fig. 1. This is the Fig 1 Title A two-dimensional plot of the PCoA of all analyzed *Centranthus* individuals.

Fig. 2. This is the Fig 2 Title. (A) Relationship between the number of determined groups (K) and LnP(D); (B) Relationship between the number of determined groups (K) and estimated ΔK; (C). Genetic relationships among the populations of *Centranthus* estimated using STRUCTURE program based on ISSR data. STRUCTURE clustering results for K = 3 to as suggested in (B).

3.2. Essential oils analysis of *Centranthus kellereri* and *C. ruber*

3.2.1. Qualitative composition of the essential oil (EO)

Statistical analyses of the 2018 data indicated that the ANOVA *p*-values for plant part were significant for the EO compounds (Table 2).

Composition of Centranthus kellereri EO in the 2018 experiment

In the 2018 experiment, the concentration of furfural in the *C. kellereri* EO from aboveground plants parts was higher in the samples from Bansko (4.9%) and lower in the samples from Vratsa (1.02%). However, furfural concentration in the roots EO was not significantly different between the plants at the two locations (populations) (5.25 and 4.86%, at Bansko and Vratsa, respectively) (Table 3).

Methylvaleric acid (isovaleric acid) was the major constituent of the EO in *C. kellereri*, ranging between 60.2% and 71.7% of the total EO, with the highest concentrations in the aboveground plant parts from Vratsa and the lowest in the roots from Vratsa. 3-Methylvaleric acid was another major constituent in the EO, ranging from 6.2% (in the biomass EO from Bansko) to 20.6% in the roots EO from Bansko, with significant differences between the locations (Table 2). Overall, the

concentration of 3-Methylvaleric acid in the EO of *C. kellereri* was higher in the aboveground plant parts than in the roots in Vratsa. However, the reverse trend was observed in the plants from Bansko, which had a higher concentration of this compound in the roots. The concentration of benzene acetaldehyde in the EO varied from undetected (in the aboveground plant parts from Vratsa) to 4.1% in the roots from the same location. Again, the reverse trend was observed in the plants collected at Bansko, which had a more abundant concentration of this compound in the aboveground plant parts than in the roots (Table 3). The concentration of borneol also varied depending on the location and the plant part, ranging from undetected amounts in the roots from Bansko to 14.3% in the aboveground parts from Bansko and 15.1% in the roots from Vratsa. Other unidentified constituents in the EO varied from 6-7% to 11-12%. The concentration of monoterpenes was highest in the aboveground parts from Bansko and in the roots from Vratsa, ranging from 2.3 to 19.2% of the total oil (Table 3).

Composition of *Centranthus ruber* root EO in the 2018 Experiment

The concentration means of the 8 EO compounds from the roots of *Centranthus ruber* are given in Table 4. Generally, the concentration of *C. ruber* EO compounds from roots was similar to their respective concentrations in *C. kellereri* roots (Table 4 and Table 5). The concentration of furfural in the root EO of the two species was similar; however the concentration of 3-methylbutanoic acid was higher in *C. ruber* than in *C. kellereri*. Furthermore, borneol was not detected in *C. ruber* root EO, similar to the *C. kellereri* EO from Bansko, while benzene acetaldehyde and the monoterpenes concentrations in root EO of *C. ruber* and *C. kellereri* from Bansko were similar. Also, the concentration of 3-methylpentanoic acid in *C. ruber* was around 10%, which

was between the concentrations of this compound in the *C. kellereri* root from the two locations, Bansko and Vratsa.

The 2019 Centranthus experiments

As indicated, in 2019, separate experiments were conducted using *Centranthus kellereri* samples from the two locations (populations), Bansko and Vratsa, as well as commercially grown *Centranthus ruber* at the experimental fields of the Institute for Roses, Aromatic and Medicinal Plants in Kazanluk. The samples collected in the 2019 experiment were from slightly different interpopulation locations compared to those in 2018, and they were collected at different timeframes and growth stages, and underwent different sample processing, such as EO isolation. Therefore, the results from the two experiments conducted in 2018 and 2019 cannot be compared.

The ANOVA *p*-values that show the significance of the effects of Location and Plant part nested in Location on 18 compounds from the 2019 Experiments are shown in Table 5. Significant effects that require multiple means comparison are shown in bold.

The concentration of 3-methylbutanoic acid in the root and aboveground plant parts EO varied significantly, ranging from 0.56% to 28%. Generally, the concentration was highest in the roots from Vratsa, followed by roots from Bansko, aboveground parts from Vratsa, and aboveground parts from Bansko, with significant differences between them. The concentration of 3-methylpentanoic acid in the EO also varied significantly, from 0.2 to 22 % of the oil, in the following order; aboveground parts Vratsa > roots Vratsa > roots Bansko = aboveground parts Bansko (Table 6).

The concentration of germacrene D in the EO varied significantly from 0.33 to 2.44% in the following order: aboveground parts Bansko > roots Bansko > roots Vratsa > aboveground parts Vratsa.

The concentration of n-tetradecanal in the EO also varied significantly from 9.9 to 18.8%, with the highest concentration of the aboveground parts from Bansko and the lowest in the aboveground parts of Vratsa.

The concentration of geranyl valerate in the EO was 1-3% of the total, with the highest in roots from Bansko and lowest in the aboveground parts from Bansko.

2Z,6E)-Farnesyl acetate concentration of the EO varied from 2.4 (aboveground parts Bansko) to 8% (aboveground parts Vratsa) of the total oil. E,E-Geranyl linalool varied from 1.5% (aboveground Vratsa) to 6.2% (roots Vratsa). Octadecatrien-1-ol (Oct-1) was one of the major EO constituents and varied from 2.8% (aboveground Vratsa) to 23.9% (aboveground Bansko). n-Octadecanol (n-Oct) concentration was between 1.6% (aboveground Vratsa) to 5.1 (roots Vratsa) (Table 6).

Octadecatrienal concentration in *C. kellereri* varied from 5.4% (aboveground parts at Bansko) to 20% (aboveground parts at Vratsa) (Table 7). Tetramethyl-2-hexadecen-1-ol varied from 1.1% (roots at Vratsa) to 7.7% (roots at Bansko) of the total EO, while 9,12-octadecadienoic acid was from 0.98% (roots Vratsa) to 8.1% (aboveground Vratsa), and 9,12,15-octadecatrienoic acid was from 0.5% (aboveground Vratsa) to 5.5% (aboveground Bansko).

Overall, the monoterpenes constituted 1.1% (aboveground Bansko) to 5% (aboveground Vratsa), sesquiterpenes were 4% (aboveground parts Vratsa) to 15.8% (roots Bansko), and long-chain alkanes were 1.3 % (aboveground parts Vratsa) to 9.5% (above ground parts at Bansko). Fatty acids constituted major part of the EO from 11.9% (above ground parts Bansko) to 39.9% (roots Vratsa) (Table 6). Other constituents (esters, alcohol, fatty aldehyde) of the EO from the 2019 Experiments were the major part of the EO with an overall concentration from 45.8% (roots Vratsa) to 64.7% (aboveground Bansko) (Table 7).

EO constituents of Centranthus ruber from 2019

The mean concentration (%) of the compounds measured in the aboveground and roots EO of *C. ruber* are provided in Table 8. Overall, 3-Methylbutanoic acid, 3-Methylpentanoic acid, and fatty acids (esters, long-chain, methil-branched) were more abundant in root EO, while germacrene D, n-tetradecanal and long-chain alkanes were more abundant in the aboveground EO of *C. ruber* (Table 8). The *C. ruber* EO from roots and aboveground plant parts showed similar abundance of monoterpenes, monoterpenoids, sesquiterpenes and sesquiterpenoids (Table 8).

Hexane extracted EO of Centranthus kellereri and C. ruber

The chemical profile of the hexane-extracted EOs from the *Centranthus kellereri* and *C. ruber* samples is shown in Table 9 and Table 10. Overall 3-methylbutanoic acid was most abundant in the roots of *C. kellereri* from Vratsa (65.9%).

Overall, 3-methylpentanoic acid was the most abundant in *C. kellereri* roots from Vratsa and Bansko, (2E)-tridecen-1-al was most abundant in the leaves and stems from Vratsa. n-Tetradecanal was the most abundant in the *C. kellereri* from Bansko, leaves and stems of *C. kellereri* in Vratsa, leaves and stems in *C. ruber*. (2E,6E)-Farnesyl acetate (2E,6E), was the most abundant in the leaves of *C. kellereri* from Vratsa, n-hexadecoic acid (n-Hex), was the most abundant in the *C. ruber* leaves and stems, and (Z,Z)-9,12-octadecadienoic acid (Z,Z) was the most abundant in the *C. kellereri* from Bansko (Table 9).

Furthermore, n-tetracosane (n-Tetrac) was the most abundant in the *C. kellereri* stems from Vratsa (25.7%) and not detected in *C. kellereri* and *C. ruber* roots.

Other compounds such as the sum of aldehydes, alcohols, ethers (Other), were most abundant in the stems and leaves of *C. kellereri* from the two locations and in *C. ruber*, with lesser amounts in the roots of the two species (Table 10). Also,

monoterpenoid (Mono) were the most abundant in the roots of *C. kellereri* from Bansko, while Sesquiterpenoids (Sesq) were most abundant in the *C. kellereri* from Bansko and in roots of *C. ruber*. Fatty acids (Fatty) were major EO constituents in all EO, most abundant in the *C. kellereri* roots EO from Vratsa, while the long-chain alkanes (Long) were most abundant in the *C. ruber* flowers (Table 10).

3.3. Embryological research

The observation on the permanent slides revealed the following peculiarities of the male and female generative sphere of the two studied species:

3.3.1. Male generative sphere

The anther is tetrasporangiate (Fig. 3A). The anther wall develops by the Dicotyledonous type according to Davis classification [40] and consists of four layers: epidermis, endothecium, one middle layer and tapetum that at the beginning of the anther ontogenesis do not differ from each other. During the meiosis in microspore mother cells (MMCs) and development of male gametophyte, the anthers layers undergo changes: the epidermal cells enlarged and rounded up outside becoming toothed, with thickened outer wall (Fig. 3 B,D); the endotecium developed fibrous thickenings after the stage of one-nucleate pollen and as result of division in tangential direction of some of them, the layer become partially two rowed (Fig. 3B); the cells of middle layer become flattened, pressed from the growing cells of the endothecium and tapetum and the layer degenerated about the second (homotypic) division of meiosis in MMCs; the tapetum differentiated into amoeboid one, atypical after the formation of unicellular pollen (Fig. 3C). Two-layered sporogenous tissue and predominantly tetrahedral microspore tetrads formed after simultaneous microsporogenesis in MMCs were observed. The mature pollen grains are 3-celled (with generative cell and two sperm) (Fig. 3B), 3- colpato-porate (Fig. 3D).

Fig. 3. This is Fig. 3 Title - Anther and development of the male gametophyte: A–tetrasporangiate anther; B – two celled mature pollen grains; C- mature pollen grains and anther wall with epidermis and fibrous endothecium; D – mature pollen grains; E –flower with anther and ovary; al - anther locule, gt –glandular trihomes, ep–epidermis, en–endothecium, al – anther locul, tp-tapetum, c-colpi, p-pori; pg-pollen gran. Magnification: A (x100); B,C,D (x400).

3.3.2. Female generative sphere

The ovary is 3-locular, but only one of the three locules is fertile (Fig. 4A). The ovule is anatropous, tenuinucellate and unitegmic (Fig. 4B). The funiculus is massive, enough long, and its epidermal cells do not form obturator within the still hemitropous ovule unicellular archesporium forms hypodermally (Fig. 4C). It differentiates directly into a megaspore mother cell (MMC) without of parietal cells formation. As result of meiosis running in MMC, a linear megaspore tetrad formed. The embryo sac (ES) development begins from the chalazal megaspore of the tetrad according to *Polygonum* (monosporic)-type. The mature ES consists of a 3-celled egg apparatus with a pyriform egg cell and two synergids (Fig. 4D), a central cell and three antipodals. The synerdgids in mature ES are pyriform and hooked. The central cell forms before the fertilization, after the fusion of the two polar nuclei located near to the antipodals that are three, situated at the chalazal end of ES, resembling in shape and structure the egg apparatus but with smaller dimensions, and no proliferating.

Fig. 4. This is the Title for Fig. 4. - Ovule and development of the female gametophyte. A. – Flower with pistil and stamen; B -Anatropous unitegmic ovule; B. Mature *Polygonum*-type embryo sac (ES); C. – *Hemitropous* ovule with unicellular archesporium; D. - Mature ES with egg apparatus and central cell; ovy-ovary, ov–ovule, f-funiculus, it–integumental tapetum (endothelium),al-anther locul, ac-archesporial cell, eggc – egg cell, syn – synergids, cc – central cell, ant – antipodal. Magnification: A, (x100); B-D (x400).The legitimate embryo and endosperm form after a porogamous double fertlization. The embryo formation follows the Asterad-type according Johansen classification (Johansen 1950). The endosperm is cellular without passing through a free nuclear stage. In the seeds it is represented from one layer around the embryo (Fig. 6E).

In *Centranthus ruber* the formation of an embryo without suspensor situated deeply in the endosperm was observed (Fig. 5B). Its topography suggests that it originates from the endosperm cells.

Fif. 5. This is the Title for Fig. 5. Development of the female gametophyte of *Centranthus ruber*. A.-Legitimate Asterad-type embryo; B-Endospermal embryo; lem-legitimate embryo, eem-endospermal embryo.

3.3.3. Pollen viability

As a result of the counting of the viable and nonviable pollen grains in the anthers of two studied species, the ratio of viable pollen was established to be 94.80±2.36% for *C. kellerei* and 92.09±2.45% for *C. ruber*.

3.3.4. Seed viability

On the base of the results of tetrazolium testing, the seeds from the two studied species were grouped in four classes (Figs. 6 and 7):

Class I: seeds with embryos stained 100% in red (12 % for the population of *C. kellereri* and 4 % for the population of *C. ruber*, Fig. 6A);

Class II: seeds with partially colored embryos (the root tip and part of cotyledons stained in red - 30 % for the population of *C. kellereri* and 10 % for the population of *C. ruber* – Fig. 6 B,C);

Class III: seeds with colorless embryos (3 % for the population of *C. kellereri* and 6 % for the population of *C. ruber* – Fig. 6D);

Class IV: empty seeds with (52 % for the two studied species-Fig. 6E).

Following the criteria [32] for interpreting the resulting coloring from tetrazolium testing, the seeds from Classes I and II were estimated as viable, and the resulting percent of viable seeds were 42 % for the population of *C. kellereri* and 14 % for the population of *C. ruber* (Fig. 7)

Fig. 6. This is the Title for Fig. 6. Estimation of seed viability according to Tetrazolium test: A – Viable, red colored embryo: B, C – Viable, partially colored embryos /B-colored root top and part of cotyledons, C – colored the root top/, stained in dark red /C/; D – Unstained unviable embryo, D- Unviable, empty seed (with seed coat and endosperm, and without embryo); end-endosperm, sc-seed coat.

Fig. 7. This is the Title for Fig. 7. Frequency of seeds (embryos) viability (%) assessed by tetrazolium test: 1 – Class I, viable embryos (stained in red); 2 – Class II,

viable embryos (partially colored embryos); 3 – Class III, unviable embryos (unstained); 4 - Class IV, unviable (empty) seeds.

3.4. Micromorphological analysis by Scanning Electron Microscopy (SEM)

The microstructure of the surface of the epidermis of the leaf, stem and the fruit of *C. kellereri* (population Bansko and Vratsa) and *C. ruber* were observed and analyzed by Scanning Electron Microscopy (SEM). Previously, it has been demonstrated that the micromorphology of the epidermis (shape and surface of the main epidermal cells; stomata; epicuticular waxes; striations) and fruit surface are important taxonomic characteristics [41, 42, 43]. These characteristics provide information for plant identification at submicroscopic level and are widely used by taxonomists to establish phylogenetic relationships [44, 45].

3.4.1. Leaves surfaces of Centranthus kellereri and C. ruber

The leaves of *C. kellereri* and *C. ruber* are simple, ovate-oblong to linear, oppositely arranged. The SEM analyses showed that the leaf surface is covered with a thick layer of epicuticular waxes. The waxes are arranged as threads and form a network between the veins of the leaf, and on the veins themselves they are in a thick and continuous layer (Fig. 8 A, B). The main epidermal cells in *C. kellereri* are isodiametric-polygonal to elongated (Fig. 8A, B), while the epidermal cells in *C. ruber* are isodiametric (Fig. 9).

According to the terminology of [46, 33] the surface of the periclinal walls of the leaves in both studied species is Convex type, with striations in *C. kellereri*. The anticlinal walls in *C. kellereri* are rounded-edged, striated, and vary from straight to S-shaped, convex [46]. The stomata are located on both epidermal surfaces (amphistomatic leaf). They are either immersed or not, depending on in which part of

the epidermal surface they are located - in the convex part or in the concave part (Fig. 8A, B).

At the edge of the stomatal closing cells of *C. kellereri*, the epicuticular waxes form minor stomatal chimneys (Fig. 8B). A significant variation in the micromorphology of perioral cells was found in this study, both between the two Centranthus species and between the two populations of *C. kellereri* (Bansko and Vratsa) (Fig. 8A, B). The cells from the stomatal complex in *C. ruber* are isodiametric with a smooth surface (Fig. 9), while in *C. kellereri* these cells are isodiametric-polygonal to elongated with striations (Fig. 8A, B).

In *C. kellereri* from Vratsa, the striations of the epidermal cells are oriented longitudinally or transversely on the cell surface (Fig. 8A), while in the *C. kellereri* from Bansko, the striations are longitudinal to the cell (Fig. 8B).

Depending on the arrangement of the striations on the cells from the stomatal complex of *C. kellereri* from Vratsa, the following varieties were established: stomatal complex where the cells were by transversely located striations (Fig. 8A); stomatal complex where the cells were surrounded by longitudinal striations (Fig. 8A); or stomatal complex where the cells do not have striations (Fig. 8A). The cellss in the stomatal complex of *C. kellereri* from the Bansko population is relatively constant and the striations are longitudinal. Single capitate glandular trichomes were found on the surface of *C. kellereri* (Fig. 8 A, B).

3.4.2. Stem surfaces of Centranthus kellereri and C. ruber

According to [13] the stem of *C. kellereri* and *C. ruber* is smooth and erect. From the SEM analyzes performed in this study, it became clear that at micromorphological level, the epicuticular waxes form longitudinal ridges that give a ribbed shape to the stem (Fig. 10). Stem waxes in *C. kellereri* differ both in shape and in arrangement. At

the base of the stem, they form a continuous waxy crust layer, which continues along the convex part of the edges along the entire length of the stem.

Further up the stem in the areas between the edges the waxy layer is thinner and crystalloids of various shapes, sizes and orientations are observed on it (Fig. 10). According to the terminology of [33], platelets and plates crystalloids were observed in this study (Fig. 10). The platelets were the most common crystaloids. The pattern of orientation of platelets was not uniform, as is the manner and angle at which they attach to the surface of the epidermis. For example, around the stomata they are locally isolated, while in other parts of the stem they are randomly oriented (Fig. 10). The wax platelets also differ in the type of their surface and their edges, which are most often irregular and wavy. Plates were also established in separate segments between platelets. The arrangement of crystalloids, their orientation and density can be used as a taxonomic feature.

3.4.3. Fruits surfaces of Centranthus kellereri and C. ruber

The micromorphological features of the fruit surfaces of *C. kellereri* and *C. ruber* analyzed by SEM are shown on Fig. 11; Fig. 12 A, B. The fruits (an achene) in both species of *Centranthus* are egg-shape elongated with a dry pericarp which is fused with the seeds. In both species, the fruits have clearly formed dorsal and ventral sides. On the dorsal side of the fruit of both species of Centranthus, 5-6 longitudinal ridges are observed, and on the ventral side there is only one ridge. In the front part of the fruit, where the sepals attach, a disc-like expansion (like a balloon) was observed. In the fruits of *C. ruber* this disc-like extension has characteristic semi-circular lobes (Fig. 11), while in *C. kellereri* this extension has no definite shape but has numerous papillae (Fig. 12 A, B). Stomata were observed on the exocarp of *C. kellereri* fruits from both populations (Bansko, Vratsa) (Fig. 12A, B).

The exocarp of the fruit of *C. ruber* is made up of cells with a rectangular rhombic shape and clearly differentiated anticlinal walls (Fig. 11). If we use the terminology of [46], the surface of the periclinal walls in the fruit of *C. ruber* is Tabular type to slightly Convex type (Fig. 11).

The cells of the exocarp in *C. kellereri* from both the Bansko and Vratsa populations are diamond-shaped and in some parts of the fruit have unclear anticlinal walls (Fig. 12 A, B). Around the edges of the fruit, the anticlinal walls in this species are significantly elevated compared to the rest of the fruit surface. If the terminology defined by [46] is used, then the surface of the periclinal walls in the fruit of *C. kellereri* is Tabular type and only around the edges it becomes slightly Convex type (Fig. 12 A, B). Papillae and short hairs were observed in fruits from both populations where the carpels fuse and in the expanded part of the margins (Figs. 12A and 12B).

3.5. Phytochemical analyses

Metabolomic analyses of the extracts from aboveground and root samples of the *Centranthus* species revealed a number of compounds (Fig. 13). Screening of extracts of the roots and aboveground plant parts against the Enzo Life Sciences Natural Products Library revealed the presence of 32 compounds that were tentatively identified as 6 simple phenolics (caffeic acid, scopoletin, chlorogenic acid, vanillyl acetone, rosmarinic acid, shikimic acid), 18 flavonoids (flavokawain A, eriodictyol-7-*O*-glucoside, marein, dihydrorobinetin, kaempferol-7-*O*-neohesperidoside, apigenin, apigenin-7-*O*-glucoside, luteolin, maritimein, isoquercitrin, eriocitrin, diosmin, narirutin, isorhoifolin, naringenin, rutin, isorhamnetin-3-*O*-glucoside, isorhamnetin-*O*-rutinoside), 1 quinone (emodin), 1 lipid (phytosphingosine), 1 alkaloid (stachydrin), 2 diterpenes (lagochiline, sclareol), and 3 triterpenoids (ursolic acid, betulinic acid, oleanolic acid). Fig. 13 shows a heatmap with the distribution of the phytochemicals

across the species, plant parts, and location. Roots and shoots differ phytochemically much more than the two species or accessions from different harvest locations (Fig. 13).

Fig. 13. This is the Title for Fig. 13. Heatmap showing distribution and relative abundance of phytochemicals in *Centranthus* spp. The red and blue colors represent abundance for each phytochemical relative to its mean set at zero. Some of the compound names are truncated due to character limitations; full names of compounds can be found in the Results section.

3.6. Antioxidant activity

Total phenols and flavonoids

The studied indicators are presented in Table 11. Overall, of the three extractants, 70% ethanol showed the strongest extraction properties for total phenolics and flavonoids, followed by water and finally acidic methanol.

The 70% ethanol extract of *Centranthus ruber* had the highest polyphenol concentration (7.976 mgGAE/gDW), followed by *Centranthus kellereri* from Bansko and Vratsa (with 6.382 and 6.331 mgGAE/gDW, respectively).

With the same extractant, the highest values for flavonoids were reported at *Centranthus kelleri* from Vratsa (302.9 mg QE/gDW), followed by *Centranthus ruber* and *Centranthus kelleri* from Bansko (226.2 and 220.7 mg QE/gDW respectively).

Free radical scavenging activity

The antiradical activity of the extracts was measured with ABTS and DPPH against the Trolox standard. The results demonstrated that aqueous and ethanol extracts in all populations show approximately the same ABTS radical scavenging activity. Only

acid methanol extracts had statistically lower activity, being lowest in the Kazanlak population (0.667 mgTE / gDW) (Table 4).

With regard to DPPH radical scavenging activity, in the *Centranthus kellereri* population of Vratsa, all three extracts gave approximately the same values, however, the values were not statistically different (0.264; 0.253; 0.235), but turn out to be higher than the results obtained in the other populations. The extracts obtained from the *Centranthus ruber* Kazanlak population showed the weakest radical scavenging activity.

4. Discussion

4.1. Genetic diversity and differentiation

In interpreting our ISSR data we found moderate levels of genetic diversity across the populations of *Centranthus* species in Bulgaria, as reflected in the values for e.g. genetic diversity and Shannon's information indices (Table 13). The prevalence of sexual reproduction and relatively good reproductive capacity explain the genetic diversity found in *Centranthus* populations. On the other hand, the restricted distribution may also affect the genetic diversity. Therefore, the observed moderate levels of genetic diversity in *Centranthus* populations could be explained by the putative effect of reduced gene flow among populations, since the habitats are separated by inter-mountains valleys and mountains. In addition, private alleles were noted (Table 13). Most probably, the existence of private alleles was due to the restricted gene flow. In our opinion, the topology of localities, as well as the geographical distance between investigated population acts as a barrier to seed and pollen dispersal. The spatial discontinuities are common in *Centranthus* populations because of their specific habitat requirements and they act simultaneously as a temporal barrier to pollen-mediated gene flow arising from a significant difference

with spatial isolation between the habitats and enhance local adaptation. Our assumptions were also supported by the PCoA (Fig. 1) and structure (Fig. 2). This geographical isolation in the *Centranthus* populations typically restricts gene flow leading to reduction of genetic diversity and increasing the differences between investigated populations. In the longer term, if the populations of the species remain small, the potential further decrease in their level of genetic diversity may cause a reduced fitness and lack of species' adaptability to the changing environments.

The maintenance of moderate diversity in *Centranthus* populations, even the presence of a significant geographic distance between populations today, probably is a result of the maintenance of high genetic diversity at a historical moment of population connectivity.

The estimate of gene flow obtained in this study (Nm = 0.899) is not high and can't prevent further differentiation through genetic drift, although, the result of AMOVA revealed that *Centranthus* populations showed some good level of within-population genetic variability (66% of their total genetic variation). This should be related to the out crossing nature of *Centranthus* species. Our findings suggest that the present-day distribution of *C. kellereri* and *C. ruber* has likely been limited to the ecological factors and habitat specialization.

Understanding genetic diversity and population structure of *C. kellereri* and *C. ruber* in Bulgaria is critical for effective management of its genetic resources. Genetic variation has increasingly been recognized as crucial to success in the long-term management of species and therefore should be a central concern for the long-term conservation. From the results revealed in our study, the levels of genetic diversity of *Centranthus* populations have reduced, we assess that the main threats are the

conditions of its habitats, population specialization, as well as the anthropogenic impact.

4.2. Embryological research

The present study is the first one on the peculiarities of the reproductive biology of Bulgarian population of *C.ruber* and *C.kellereri* is a contribution for the embryological characteristic of genus *Centranthus*.

The most of the revealed in the study characteristics of the structures in male and female gametophytes are in accordance with the described ones for the representatives of Valerianaceae family that in the actual Plant List [47]. is placed as subfamily in the Caprifoliaceae family, and namelly: tetrasporangiate anthers, 4-layered anther walls, ameboid tapetum, normal meiosis of the simultaneous type, anatropous tenuinucellate ovule, *Polygonum*-type development of female gametphyte with hooked synergids and three noproliferating antipodal cells with arrangement similar to this of cells of egg apparatus, Asterad-type of embryo formation [48, 49, 50].

The established in *C. ruber* endospermal embryo is evidence of higher plasticity of the female gametophyte, which provides greater adaptability to environmental changes and, correspondingly, stability of the populations of the species. It is a new feature for genus *Centranthus*. For this genus only formation of endospermal haustorium in *C.calcitrapa* was reported by [50] and for Valerianaceae family- a poliembriony in genus Valeriana by the same authors.

[51] comparing the germination ecology of *C. ruber* and the endemic for Sardinia *C. amazonum* established lower seed germination percentage and more empty seeds in *C. amazonum* than in *C. ruber*. The results of present study revealed the contrary: a higher percentage of seed viability was established in the seeds of *C. kellereri* (Bulgarian endemic). This fact is the comprobation of the reported also in species

from other families, dependence of seed quality and respectively germination of the environmental conditions. For example, [52] established that the seed germination behavior in two endemic species of the genus *Sideritis* was closely related to the characteristics of habitats, mainly the environmental conditions (temperature and sunlight intensity) and soil structure. Generally, the established low (12 % in *C. ruber*) to moderate (42 % in *C. kellereri*) seed viability is one of causes for limited distribution of two studied species in Bulgaria.

4.3. Morphological analysis by Scanning Electron Microscopy (SEM)

Centranthus kellereri is an endemic plant species found only in Bulgaria [53], classified as Critically Endangered ([CR B2ab(v)]). However, the micromorphological features of its leaves, stems, and fruits have not been previously studied. Therefore, this is the first study to describe the characteristics of the epicuticular waxes present on the leaves and stems of this species. Furthermore, the study also revealed the characteristics of the exocarp in the fruits of *C. kellereri* and compared them with those in *C. ruber*. The morphological characteristics of the flowers, fruits, microscopic structure of the epidermis (on the leaf, stem, and fruits), and pollen in species belonging to the genus Centranthus, are of taxonomic significance [54, 55, 56].

These traits are characterized by a high degree of variability [55, 56, 57, 58, 59], which is a prerequisite for the accumulation of taxonomic synonymy and interpretations. The SEM analysis of the leaf epidermis of the two *Centranthus* species showed similarities but also significant differences in micromorphological characteristics. Differences in the orientation and striations of the cells of the stomatal complex in *C. kellereri* were set between samples from the two populations (Bansko and Vratsa).The micromorphology of the stomatal complex cells of *C. kellereri* from Vratsa was characterized by a greater diversity, which defines four types of the

stomatal complex cells. Stem epicuticular waxes in *C. kellereri* also showed diversity, in the two populations, (Bansko and Vratsa), both in shape and arrangement. *Centranthus kellereri* is known as a calciphyte and spreads on mobile stony screes (Calcareous screen) characterized by a low amount of surface water (sclerophyte). Most probably, the formation of a thick layer of the crust of epicuticular waxes and crystalloids is a reaction of the species to unfavorable conditions. Normally, plants react by synthesizing large amounts of epicuticular waxes to protect themselves from high light intensity and insufficient water.

The fruits of both species of *Centranthus* are ovoid elongated with a pappus-like disc-like extension and are morphologically similar to the fruit of *Valeriana* sp. Indeed, the genus *Centranthus* was included in tribe *Valerianeae*, subtribe *Centranthinae* Graebn. in *Valerianaceae* family. Although according to the recent taxonomic developments (APG III, 2009; APG IV, 2016) the family is referred to the family *Caprifoliaceae* Juss. (sensu lato). Papillae, short hairs and stomata were found on the surface of *C. kellereri*, which have also been found in other *Centranthus* species [60].

4.4. Essential oils composition of *Centranthus kellereri* and *C. ruber*

Although no studies on the composition of *C. kellereri* EO could be found, some have been conducted on *C. ruber*. In a study by [10], aboveground plant parts of *C. ruber* from Southern Italy were extracted with methanol via maceration, and the crude extracts were subsequently separated using solvents with increasing polarity. The dried extracts were suspended in a solution of 9:1 methanol and water, and then extracted with n-hexane. The dried residue was extracted with dichloromethane. The authors reported the presence of chemical compounds in the n-hexane and dichloromethane fractions. Fatty acids (FA) were found to be abundant in the n-hexane fraction, with palmitic acid being the most abundant (6.87%). The GC

analyses also identified four terpenes, including the monoterpene lactone dihydroactinidiolide and the isoprenoid ketone phytone (hexahydrofarnesyl acetone), and the monoterpenoid 2,6-di-tert-butyl-1,4-benzoquinone, with phytone being the most abundant at 2.8%. Six compounds were identified in the dichloromethane fraction of *C. ruber*, with benzoic acid being the most abundant (1.2%), and the rest present in trace amounts, mostly below 0.5% [10].

4.5. Phytochemical analyses

The KnapSack database [61] of plant species and their phytochemicals reveals that only three species of *Centranhus* have been characterized phytochemically: *C. macrosiphon*, *C. longiflorus*, and *C. ruber*. Our plant metabolomics approach did not detect the previously reported acevaltrate [62] in the one accession of *C. ruber* because this phytochemical is not present in our Enzo library of plant metabolites. The study focused on valepotriates and the author did not report the phytochemicals we detected in this study. Consistent with finding reported by [63] for *C. ruber*, we detected quercetin, rutin, kaempferol, luteolin, and/or their *O*-glycosides in the shoots of this plant.

4.6. Antioxidant activity of *Centranthus kellereri* and *C. ruber*

In a previous study, *Centranthus longiflorus* was shown to possess sedative and anticonvulsant effects, which were analogous to those exhibited by diazepam [18]. The genus *Centranthus* belongs to the Valerianaceae family. Usually, these species are utilized for their sedative properties [64, 65, 66]. However, the information on the chemical composition of the roots is scarce and is mainly related to the iridoids characteristic of this genus and in particular valepotriate. There is almost no

information concerning antioxidant activity and chemical content of *Centranthus kellereri*, which is Bulgarian endemic species.

This study compared two species, *Centranthus ruber*, which is widespread, and the Bulgarian endemic species *Centranthus kellereri*, from the only two existing populations in the world. The healing properties of wild plants are mainly due to their accumulated secondary metabolites, which, in turn, depend on the environmental conditions in which the plants grow. As expected, the results obtained from the studied populations differed significantly.

The method of extraction of biologically active substances is also of great importance. The use of different solvents, extraction times, or environmental conditions can significantly affect the results. In the present study, it was observed that ethanol and aqueous extracts had the highest values for phenolic and flavonoid compounds. Most of the extracts used for medical purposes are obtained with ethanol or water [64, 67] Regarding aqueous extracts, similar results have been reported by other authors who compared the effect of solvents on the content of phenols and flavonoids in *Centranthus longiflorus* [68].

Polyphenols are prominent due to their antioxidant activity and protective properties. The ethanolic extract exhibited the highest total phenolic content (7.976 mg GAE/g DW) in *C. ruber*. Conversely, methanolic extract (0.585 mg GAE/g DW) showed the lowest phenolic content in *C. ruber*. The last finding is in contradiction to the widely spread opinion for methanol is an effective solvent for the extraction of antioxidants [69].

Flavonoids show several antioxidants, antiviral, and antimutagenic effects. For example, quercetin is a well-known plant-derived flavonoid that may have anti-inflammatory and antioxidant properties. Our results on flavonoid content were

highest in *Centrantus kellereri,* Vratsa, compared to the other populations, and that was observed for all extracting solutions in the following order: ethanol>water>acidic methanol (302.9; 208.6; 156.2 mgQE/gDW). In addition, the results were higher than that previously reported in *Centranthus longiflorus* [68].

DPPH radical (2,2-Diphenyl-1-picrylhydrazyl) is one of the few stable organic nitrogen radicals, which has a deep purple color. Unlike ABTS, it does not have to be generated before the assay. The DPPH assay is mainly based on an electron transfer reaction and hydrogen-atom abstraction. This assay is based on the measurement of the reducing ability of antioxidants toward DPPH [68]. *C. kellereri* Vratsa population showed the highest radical scavenging activity in all extracts, which did not differ significantly, and were close to those obtained from the *C. kellereri* Bansko population. The population of *C. ruber* showed significantly lower scavenging activity. Interestingly, the reported results for methanolic extracts did not correspond to those obtained from other authors [68].

The scavenging ability of the ABTS radical was found to be higher than that of the DPPH radical. Surprisingly, the water extracts obtained the highest ABTS radical scavenging activity in the order regarding the populations: Vratsa>Kazanlak>Bansko.

This is the first study on the antioxidant activity of different extracts from *Centranthus kellereri*. Different extracts (ethanolic, acidic methanol, and water) were analyzed in order to obtain the total phenolics, flavonoids, and radical scavenging activities.

Given the global popularity of the closely related species *C. ruber* as a landscaping plant [70, 71] and the exceptional ability of the endemic species *C. kellereri* to thrive in inhospitable rocky screeds, there is significant potential for the endemic species *C. kellereri* to serve not only as a medicinal resource but also as a highly valuable addition to landscaping projects. Its inclusion in landscaping designs can contribute to

the creation of visually appealing and sustainable landscapes, capable of withstanding challenging conditions.

Furthermore, the utilization of *C. kellereri* in landscaping projects not only adds aesthetic value but also offers an opportunity to conserve and showcase the endemic flora, highlighting its ecological significance.

Considering these factors, it is evident that the endemic species *C. kellereri* holds tremendous promise, both in terms of medicinal applications and as a valuable asset in landscaping endeavors. Exploring and promoting its use in these domains can foster biodiversity conservation and present novel possibilities for sustainable landscaping practices.

5. Conclusions

This is the first comprehensive study on *Centranthus kellereri*, an endemic plant found only in two locations in the world.

Genetic analyses have shown a clear differentiation between *C. kellereri* and *C. ruber*, and between the two populations of *C. kellereri*. Phytochemical analyses have revealed both similarities and noteworthy differences in the content and composition of essential oils (EO) between *C. kellereri* and *C. ruber*, as well as within the two populations of *C. kellereri*. These differences include significant variations in the composition and concentration of individual compounds, which are influenced by factors such as plant part and timing of sample collection.

Further metabolimic analyses identified the presence of 32 compounds, with many shared between the shoots and roots of both *C. kellereri* and *C. ruber*. Furthermore, variations in the detected compounds were observed between the *C. kellereri* plants at different locations, as well as between the roots and shoots.

This study represents the first investigation into the antioxidant activity of various extracts derived from *C. kellereri*. Our analysis included ethanolic, acidic methanol, and water extracts, aiming to determine the total phenolic and flavonoid content, as well as the radical scavenging activities. The *C. kellereri* Vratsa population exhibited the highest radical scavenging activity across all extracts. Notably, the scavenging activities did not exhibit significant differences and were comparable to those obtained from the *C. kellereri* Bansko population. In contrast, the C. ruber population displayed significantly lower radical scavenging activity when compared to both *C. kellereri* populations. These findings shed light on the antioxidant potential of *C. kellereri* and suggest that the Vratsa population, in particular, holds promise for further exploration. The results also highlight the need for additional research to understand the underlying factors contributing to the observed differences in antioxidant activity among these populations.

In terms of the established peculiarities of the structures and processes in the male and female gametophytes of two studied *Centrantus* species, it may be defined the following conclusions:

The observed characteristics define the *Centranthus kellereri* as sexual reproducing.

- The estimated normal running of processes leading to pollen and seed formation without deviation and degenerations and high pollen viability afford *C. kellereri* a high potential for realization of its reproductive capacity that is the prerequisite for regeneration and stability of their populations.
- The observed low seed viability indicates the *C. kellereri*’s high sensitivity to environmental conditions, which serves as a crucial factor affecting population size. Therefore, it is recommended to monitor the populations of *C. kellereri*,

establish a seed bank, and implement experimental ex situ collections to propagate and reinforce their natural populations as needed.

The SEM analyses revealed notable variations in surface microstructural traits not only between the species (*C. kellereri* and *C. ruber*) but also within the two populations of *C. kellereri*. Consequently, the observed dissimilarities in genetic makeup, micromorphological characteristics, and phytochemical composition strongly indicate that the two populations can be classified as distinct subspecies or forms (or varieties) of *C. kellereri*; var. *Pirinensis* and var. *Balkanensis*

These findings underscore the importance of recognizing and differentiating between these populations, as they likely possess unique biological and ecological attributes. Further research is warranted to elucidate the specific characteristics and potential implications of these subspecies/forms/varieties in the context of their taxonomy, conservation, and utilization.

Considering the widespread use of the closely related species *C. ruber* as a landscaping plant worldwide and the fact that the endemic species *C. kellereri* thrives in rocky screes under extremely challenging environmental conditions, there is a strong potential for the endemic species *C. kellereri* to be utilized not only for medicinal purposes but also as a valuable landscaping plant.

Fig. 8A. This is the title for Fig 8A. Fig. 8A. Scanning Electron Microscopy (SEM) analysis of leaves surfaces of *C. kellereri* from Vratsa (V); ad – adaxial surfaces; ab – abaxial surfaces; view of trichomes, epicuticular waxes, stomata and striations.

Fig.8B. This is the Title for Fig. 8B. Scanning Electron Microscopy (SEM) analysis of leaves surfaces of *C. kellereri* from Bansko (P); ad – adaxial surfaces; ab – abaxial surfaces; view of trichomes, epicuticular waxes, stomata, striations and stomatal chimneys.

Fig.9. This is the Title for Fig. 9. Scanning Electron Microscopy (SEM) analysis of leaves surfaces of of *C. ruber* from Kazanlak (K); ad – adaxial surfaces; ab – abaxial surfaces; view of epicuticular waxes and stomata.

Fig.10. This is the Title for Fig. 10. Scanning Electron Microscopy (SEM) analysis of stem surfaces of *C. kellereri* from Bansko (P) and Vratsa (V). View of waxes, platelets and plates.

Fig.11. This is the Title for Fig. 11. Scanning Electron Microscopy (SEM) analysis of fruits surfaces of *C. ruber* from Kazanlak (K). General view of exocarp of fruits.

Fig.12A. This is the Title for Fig. 12 A. Scanning Electron Microscopy (SEM) analysis of fruits surfaces of *C. kellereri* from Bansko (P). General view of exocarp of fruits and trichomes, papili, stomata.

Fig.12B. This is the Title for Fig. 12B. Scanning Electron Microscopy (SEM) analysis of fruits surfaces of *C. kellereri* from Vratsa (V). General view of exocarp of fruits and trichomes, papili, stomata.

Acknowledgments: This research has been supported by Oregon State University startup funds awarded to Dr. Valtcho D. Jeliazkov (Zheljazkov)

Author Contributions: **Valtcho D. Zheljazkov**: Conceptualization, Methodology, Collection, Investigation, Writing – original draft, review & editing, Data curation. **Ivanka B. Semerdjieva**: Methodology, Investigation, Writing –review & editing. **Daniela Borisova**: Methodology, Collection, Investigation, Writing –review & editing. **Elina Yankova-Tsvetkova**: Methodology, Investigation, Visualization, Writing – review & editing. **Lyubka H. Koleva-Valkova**: Methodology, Investigation, Visualization, Writing – review & editing. **Galya Petrova**: Methodology, Investigation, Visualization, Writing – review & editing. **Ivayla Dincheva**: Methodology, Investigation, Writing – review & editing. **Fred Stevens**: Methodology, Investigation, Visualization, Writing – review & editing. **Wenbin Wu**: Methodology, Investigation, Visualization, Writing – review & editing. **Tess Astatkie**: Software, Investigation, Statistical analyses, Visualization, Writing – review & editing. **Tanya Ivanova**: Methodology, Investigation. **Albena Stoyanova**: Methodology, Investigation, Writing – review & editing. **Anatoli Dzhurmanski**: Methodology, Investigation.

Conflict of Interest: The authors declare that the research was conducted in the absence of any commercial or financial relationships that could be construed as a potential conflict of interest.

References

1. Velchev V. Vegetation of the Vrachanska Mt. Bulg. Acad. Sci. Press, Sofia (in Bulgarian); 1971.
2. Stojanoff N. Thracische und mazedonische Herbarmaterialen des Verstorbenen Prof. Dr. Theodor Nikoloff. – Dokl. Bulg. Acad. Sci. 1928;37(18):49-209.
3. Davies CE, Moss D, Hill MO. EUNIS Habitat Classification revised. Report to the European Topic Centre on Nature Protection and Biodiversity, European Environment Agency. October 2004. 310 pp.
4. Tzonev R, Dimitrov M, Roussakova V. Syntaxa according to the Braun Blanquet approach. Phytologia Balcanica. 2009;15(2).
5. Watson L, Macfarlane TD, Dallwitz MJ. The grass genera of the world: descriptions, illustrations, identification, and information retrieval; including synonyms, morphology, anatomy, physiology, phytochemistry, cytology, classification, pathogens, world and local distribution, and references. Version: 5th November 2021. https://www.delta-intkey.com/grass/ index.htm (accessed 17 June 2023).
6. Chami MC, Bouju E, Lequemener C, de Vaumas R, Hadji-Minaglou F, Fernandez X et al. Purification of two valepotriates from Centranthus ruber by centrifugal partition chromatography: From analytical to preparative scale. J Chromatogr A. 2018;1580(14):126-133. doi: 10.1016/j.chroma.2018.10.044.
7. Di Martino L, Di Cecco V, Di Cecco M, Di Santo M, Ciaschetti G, Marcantonio G. Use of native plants for ornamental purposes to conserve plant biodiversity: case of study of Majella National Park. J Nature Conserv. 2020;56:125839. https://doi.org/10.1016/j.jnc.2020.125839.

8. Doyle AM, Reilly J, Murphy N, Kavanagh PV, O'Brien JE, Walsh MS et al. Nature's Sedative: Isolation and Structural Elucidation of Valtrate from Centranthus ruber. J Chem Educ. 2004;81(10):1486. doi: 10.1021/ed081p1486.
9. Honma T, Shiratani N, Banno Y, Kataoka T, Kimura R, Sato I et al. Seeds of Centranthus ruber and Valeriana officinalis Contain Conjugated Linolenic Acids with Reported Antitumor Effects. J Oleo Sci. 2019;68(5):481-491. doi: 10.5650/jos.ess19007.
10. Musolino V, Marrelli M, Perri MR, Palermo M, Gliozzi M, Mollace V et al. Centranthus ruber (L.) DC. and Tropaeolum majus L.: Phytochemical Profile, In Vitro Anti-Denaturation Effects and Lipase Inhibitory Activity of Two Ornamental Plants Traditionally Used as Herbal Remedies. Molecules. 2023;28(1):32. doi: 10.3390/molecules28010032.
11. Geerts S, Rossenrode T, Irlich U, Visser V. Emerging Ornamental Plant Invaders in Urban Areas—Centranthus ruber in Cape Town, South Africa as a Case Study. Invasive Plant Science and Management. 2017;10(4):322-331. doi: 10.1017/inp.2017.35.
12. Holmes PM, Rebelo AG, Irlich UM. Invasive potential and management of naturalized ornamentals across an urban environmental gradient with a focus on Centranthus ruber. Bothalia. 2018;48(1):a2345. doi: 10.4102/abc.v48i1.2345.
13. Delipavlov D, Popova M, Ančev M. Centranthus DC. In: Kožuharov S (ed.): Florae Reipublicae Bulgaricae, Vol. 10. Editio Acad. “Prof. Marin Drinov”, Serdicae, pp. 394–396 [in Bulgarian]; 1995.
14. Tsymbalyuk ZM, Ivanova D, Nitsenko LM. Taxonomic significance of palynomorphological characteristics of selected Centranthus (Caprifoliaceae) species. Hacquetia. 2020;20(2):243–256.

15. Newman DJ, Cragg GM. Natural products as sources of new drugs over the nearly four decades from 01/1981 to 09/2019. J Nat Prod. 2020;83:770–803. doi: 10.1021/acs.jnatprod.9b01285.

16. Bertschinger L, Baur R, Carlen C, Doruchowski G. Sustainability of Horticulture in Europe (Environmental, Social, Economic): Examples from the Pre- and the Post-Harvest Food Chain. Acta Hortic. 2009;817:187-194. doi: 10.17660/ActaHortic.2009.817.18.

17. Zheljazkov VD, Craker, LE. Overview of Medicinal and Aromatic Crops. Chapter 1. In: Medicinal and Aromatic Crops: Production, Phytochemistry, and Utilization. 2016;1-12. DOI: 10.1021/bk-2016-1218.ch001

18. Buyukokuroglu ME, Demirezer LO, Guvenalp Z. Anticonvulsant and behavior-modifying effects of Centranthus longiflorus ssp. longiflorus: a study of comparison to diazepam. Pharmazie. 2002;57(8):559-61.

19. Zheljazkov VD, Kacaniova M, Dincheva I, Radoukova T, Semerdjieva IB, Astatkie T, et al. Essential oil composition, antioxidant and antimicrobial activity of the galbuli of six juniper species. Ind Crops Prod. 2018;124:449-458.

20. Adams R. Identification of essential oil components by gas chromatography/mass spectrometry. 4th ed. Carol Stream, IL, USA: Allured Publ., Corporation; 2007.

21. National Institute of Standards and Technology, USA. [Online]. Available from: https://www.nist.gov/srd [Accessed January 20, 2023].

22. Zheljazkov VD, Semerdjieva IB, Stevens JF, Wu W, Cantrell CL, Yankova-Tsvetkova E et al. Phytochemical Investigation and Reproductive Capacity of the Bulgarian Endemic Plant Species Marrubium friwaldskyanum Boiss. (Lamiaceae). Plants. 2022;11(1):114. https://doi.org/10.3390/plants11010114

23. Pang Z, Chong J, Zhou G, de Lima Morais DA, Chang L, Barrette M et al. MetaboAnalyst 5.0: narrowing the gap between raw spectra and functional insights. Nucleic Acids Res. 2021;49(W1):W388-W396. doi: 10.1093/nar/gkab382.
24. Doyle JJ, Doyle JL. A rapid DNA isolation procedure for small quantities of fresh leaf tissue. Phytochem Bull. 1987;19:11-15.
25. Peakall R, Smouse PE. GenAlEx 6.5: genetic analysis in Excel. Population genetic software for teaching and research—an update. Bioinformatics. 2012;28(19):2537–2539. doi: 10.1093/bioinformatics/bts460.
26. Pritchard JK, Stephens M, Donnelly P. Inference of population structure using multilocus genotype data. Genetics. 2000;155(2):945-959. doi: 10.1093/genetics/155.2.945.
27. Evanno G, Regnaut S, Goudet J. Detecting the number of clusters of individuals using the software STRUCTURE: a simulation study. Mol Ecol. 2005;14(8):2611-2620. doi: 10.1111/j.1365-294X.2005.02553.x.
28. Earl DA, vonHoldt BM. STRUCTURE HARVESTER: a website and program for visualizing STRUCTURE output and implementing the Evanno method. Conserv Genet Resour. 2012;4(2):359-361. doi: 10.1007/s12686-011-9548-7.
29. Romeis B. Mikroskopische Technik, 15th ed. R. Oldenbourg Verlag. München; 1948.
30. Heidenhain R. Eine Abänderung der Färbung mit Hematoxylin und chromsäuren Salzen. A.m.A. 1886;27:383-384.
31. Peters J. Tetrazolium testing handbook. Contribution №29 to the handbook on seed testing revised. The Association of Official Seed Analysts (AOSA); 2000.

32. Moore RP. Handbook on tetrazolium testing. International Seed Testing Association; Zurich, Switzerland; 1985
33. Barthlott W, Neinhuis C, Cutler D, Ditsch F, Meusel I, Theisen I et al. Classification and terminology of plant epicuticular waxes. Bot J Linn Soc. 1998;126(3):237-260. doi: 10.1111/j.1095-8339.1998.tb02529.x.
34. Singleton V, Rossi J. Colorimetry of Total Phenolic Compounds with Phosphomolybdic-Phosphotungstic Acid Reagents. Am J Enol Vitic. 1965;16:144-158.
35. Zhishen J, Mengcheng T, Jianming W. The determination of flavonoid contents in mulberry and their scavenging effects on superoxide radicals. Food Chem. 1999;64:555-559. doi: 10.1016/S0308-8146(98)00102-2.
36. Brand-Williams W, Cuvelier ME, Berset CLWT. Use of a Free Radical Method to Evaluate Antioxidant Activity. LWT-Food Sci Technol. 1995;28:25-30. doi: 10.1016/S0023-6438(95)80008-5.
37. Xiao F, Xu T, Lu B, Liu R. Guidelines for antioxidant assays for food components. Food Front. 2020;1:60–69. doi: 10.1016/j.foodfr.2020.02.002.
38. SAS Institute Inc. SAS/STAT® 9.4 User’s Guide. Cary, North Carolina, USA: SAS Institute Inc.; 2014.
39. Montgomery DC. Design and analysis of experiments. 10th ed. New York, USA: Wiley; 2020.
40. Davis G. Systematic embryology of the angiosperms. New York: J. Wiley & Sons; 1966.
41. Metcalfe CR, Chalk L. Anatomy of the Dicotyledons, 2nd Ed. Oxford Science Publications. 1979;1:96–117.

42. Barthlott W. Epidermal and seed surface characters of plants: Systematic applicability and some evolutionary aspects. Nordic J Bot. 1981;1(3):345–355. doi: 10.1111/j.1756-1051.1981.tb00704.x.
43. Evert RF, Eichhorn SE. Esau’s plant anatomy, 3rd ed. Hoboken: Wiley; 2006.
44. Barthlott W. Scanning electron microscopy of the epidermal surface in plants. In: Claugherd (ed.), Scanning Electron Microscopy in Taxonomy and Functional Morphology. 1990:69-94. Clarendon, Oxford.
45. Payne WW. Stomatal Patterns in Embryophytes: Their Evolution, Ontogeny and Interpretation. Taxon. 1979;28:117–132.
46. Barthlott W, Ehler N. Raster-Elektronenmikroskopie der Epidermis-Oberflächen von Spermatophyten. Trop Subtrop Pflanzenwelt. 1977;19:367-467. Wiesbaden: Steiner.
47. The Plant List (2013). Version 1.1. Published on the Internet. Available from:
48. Watson L, Dallwitz MJ. Valerianaceae Batsch. In: The Families of Flowering Plants: Descriptions, Illustrations, Identification, and Information Retrieval. Version: 14th December 2000. Available from: http://biodiversity.uno.edu/delta/.
49. Poddubnaya–Arnoldi VA. Characteristics of Angiosperms families according to cytoembryological features. Nauka, Moskow; 1982. 167 p. (in Russian).
50. Kamelina OP, Zhukova GYa. Valerianaceae family Davidiaceae-Asteraceae. Л. С. 1987;171–177 (In Russ.).
51. Mattana E, Daws MI, Bacchetta G. Comparative germination ecology of the endemic Centranthus amazonum (Valerianaceae) and its widespread congener Centranthus ruber. Plan Species Biology. 2010;25(3):165-172. doi: 10.1111/j.1442-1984.2010.00280.x.

52. Estrelles E, Güemes J, Riera J, Boskaiu M, Ibars AM, Costa M. Seed germination behaviour in Sideritis from different Iberian habitats. Not Bot Hort Agrobot Cluj. 2010;38:9-13.

53. Meshinev T. Centranthus kellereri (Stoj., Stef. & T.Georgiev) Stoj. & Stef. (254-255). In: Petrova A, editor. Atlas of Bulgarian endemic plants. Gea-Libris Ltd., Sofia; 2006.

54. Hidalgo O, Mathez J, Garcia S, Garnatje T, Pellicer J, Valles J. Genome Size Study in the Valerianaceae: First Results and New Hypotheses. Hindawi Publishing Corporation, Journal of Botany. 2010;19 pages. doi: 10.1155/2010/797246.

55. Al-dabbagh STS, Saeed JF. Morphological and anatomical variations of fruits in some taxa of Valerianaceae Batsch family [Special issue]. Iraqi Journal of Agricultural Sciences. 2020;51:101–115. doi: 10.36103/ijas.v51iSpecial.887.

56. Tsarenko O, Shikhaleyeva G, Minarchenko V, Tymchenko I, Bulakh O. Micromorphological Characteristics of Fruit Surfaces of Some Usable Species of the Genus Valeriana (Valerianaceae) Among Ukrainian Flora. Acta Agrobotanica. 2021;74:ID:7414. doi: 10.5586/aa.7414.

57. Ernet D. Fruchtbau und Verbreitungsbiologie von Valerianella und Fedia (Valerianaceae). Plant Syst Evol. 1978;130:85–126.

58. Xena de Enrech N, Mathez J. Re´vision du genre Fedia Gaertnemend. Moench. (Valerianaceae). Nat Monspel Ser Bot. 1990;54:3–77.

59. Xena de Enrech N, Mathez J. Genetic control of fruit polymorphism in the genus Fedia (Valerianaceae) in the light of dimorphic and trimorphic populations of F. pallescens. Plant Syst Evol. 1998;210:199–210.

60. Jacobs B, Bell Ch, Smets E. Fruits and Seeds of the Valeriana Clade (Dipsacales): Diversity and Evolution. Int J Plant Sci. 2010;171(4):421–434. doi: 10.1086/651243.

61. Afendi FM, Okada T, Yamazaki M, Hirai-Morita A, Nakamura Y, Nakamura K et al. KNApSAcK family databases: integrated metabolite–plant species databases for multifaceted plant research. Plant Cell Physiol. 2012;53(2):e1-e. doi: 10.1093/pcp/pcr165.

62. Hölzl J. Bedeutung von chemischen rassen, herkünften, cultivars und arten für die in–vivo präparation radioaktiv markierter naturstoffe, dargestellt am beispiel von Valeriana. Planta medica. 1975;28(07):301-14.

63. Pagani F. Phytoconstituents of some *Centranthus* (*Valerianaceae*) species found in Liguria. Bollettino Chimico Farmaceutico. 1981;120(8):463-8.

64. Murray, MT. *Valeriana officinalis* (Valerian), Book chapter 123 - in Textbook of Natural Medicine (Fifth Edition), 2020 (1)4;902-905.e1, Imprint Churchill Livingstone, ISBN: 978-0-323-52342-4, https://doi.org/10.1016/C2015-0-02243-2

65. Buysse DJ, Tyagi S, Chapter 42 - Clinical Pharmacology of Other Drugs Used as Hypnotics in Principles and Practice of Sleep Medicine (Sixth Edition), 2017; 432-445.e7, https://doi.org/10.1016/B978-0-323-24288-2.00042-8

66. Gulati K, Anand R, Arunabha Ray A. Nutraceuticals as Adaptogens: Their Role in Health and Disease. Nutraceuticals, Efficacy, Safety and Toxicity, Chapter 16 - 2016;193-205, https://doi.org/10.1016/B978-0-12-802147-7.00016-4

67. Buysse, DJ, Morin CM, Hypnotics in Encyclopedia of the Neurological Sciences, 2003;612-614, https://doi.org/10.1016/B0-12-226870-9/00251-3

68. Zengin G, Nithiyanantham S, Locatelli M, Ceylan R, Uysal S, Aktumsek A et al. Screening of In vitro Antioxidant and Enzyme Inhibitory Activities of Different Extracts from Two Uninvestigated Wild Plants: Centranthus longiflorus subsp. longiflorus and Cerinthe minor subsp. auriculata. Eur J Integr Med. 2016;8:286-292. doi: 10.1016/j.eujim.2015.12.004.
69. Hertog MG, Feskens EJ, Hollman PC, Katan MB, Kromhout D. Dietary antioxidant flavonoids and risk of coronary heart disease: the Zutphen elderly study. Lancet. 1993;342:1007-1011. doi: 10.1016/0140-6736(93)92876-U.
70. Benvenuti S, Malandrin V, Prdossi A. Germination ecology of wild living walls for sustainable vertical garden in urban environment. Scientia Horticulturae. 2016;203:185-191. doi: 10.1016/j.scienta.2016.03.031.
71. Kratsch H, Heflebower R. Gardening Guide for High-Desert Urban Landscapes of Great Basin Regions in Nevada and Utah. University of Nevada Cooperative Extension. SP-13-09.

Figure1

Click here to access/download;Figure;Fig.1.tiff

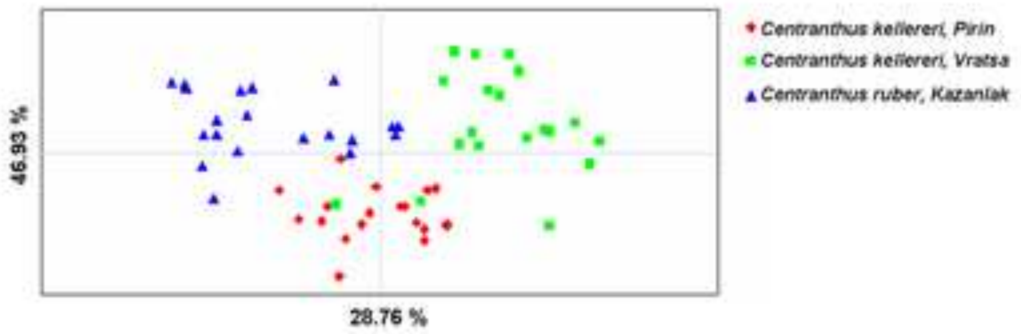

Figure2

Click here to access/download;Figure;Fig2.tiff

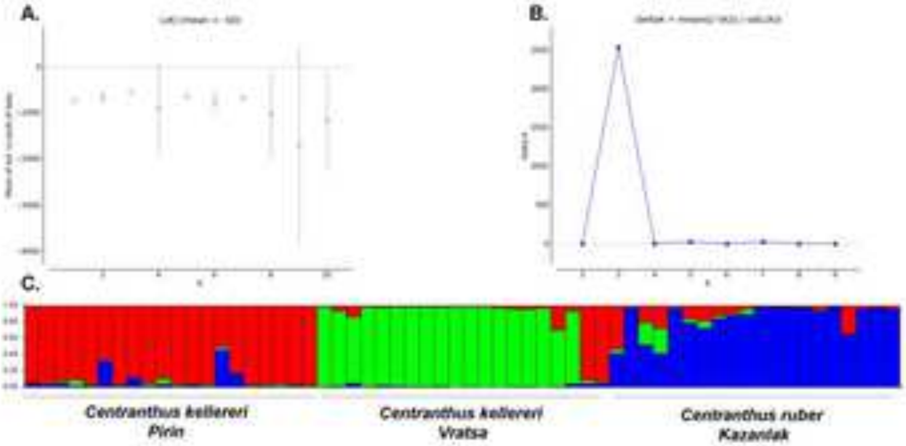

 Click here to access/download;Figure;Fig.3.tiff

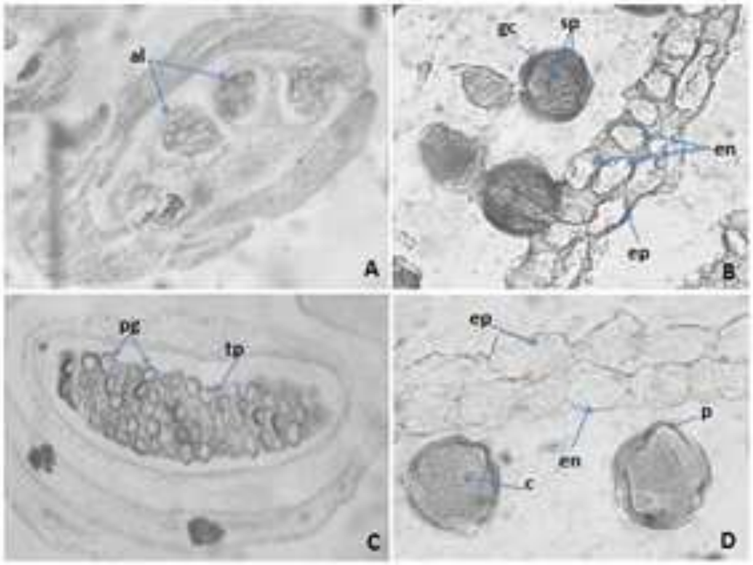

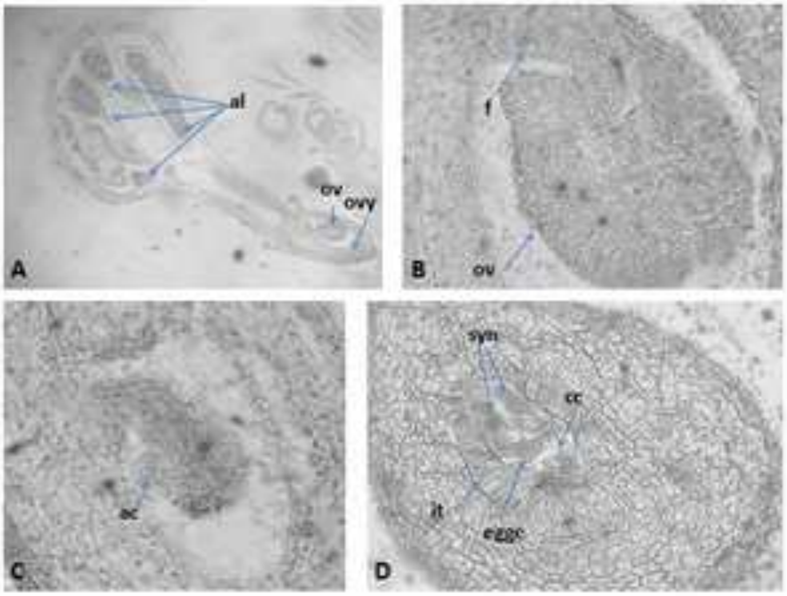
al
ov
ovy
A
f
ov
B
ac
C
syn
cc
it
eggc
D

Figure5

Click here to access/download;Figure;Fig.5.tiff

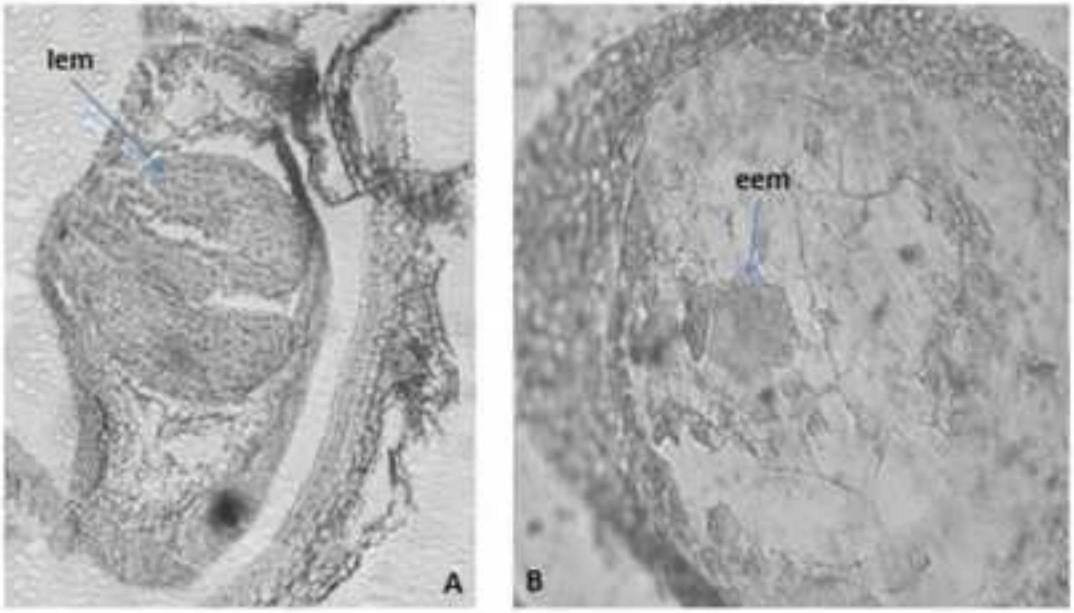

Figure6

Click here to access/download;Figure;Fig.6.tiff

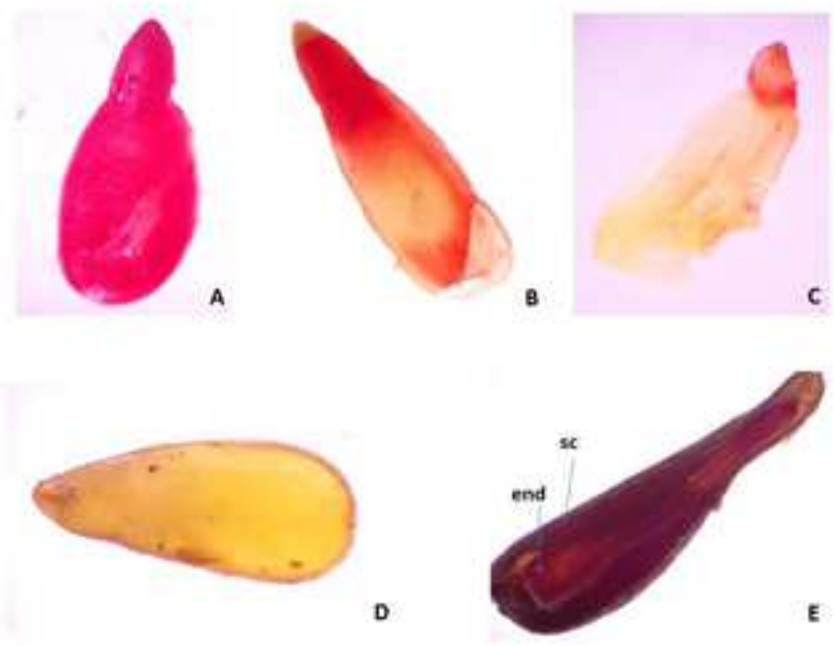

Figure7

Click here to access/download;Figure;Fig.7.tiff

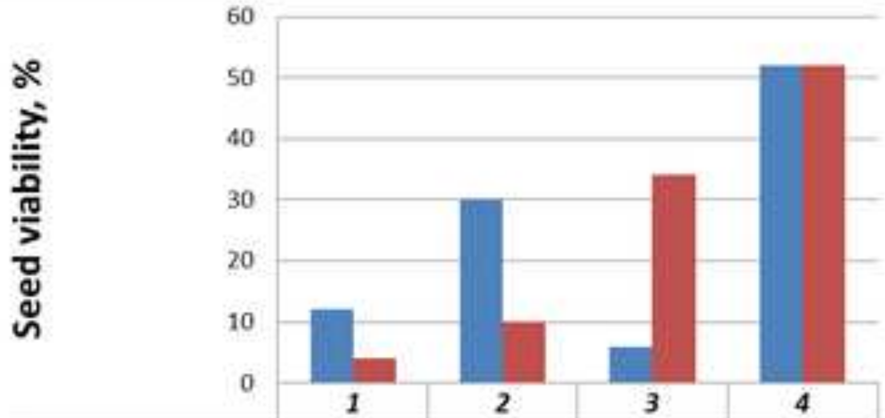

	1	2	3	4
Centranthus kellereri	12	30	6	52
Centranthus ruber	4	10	34	52

Figure8A

Click here to access/download;Figure;Fig.8A.tiff

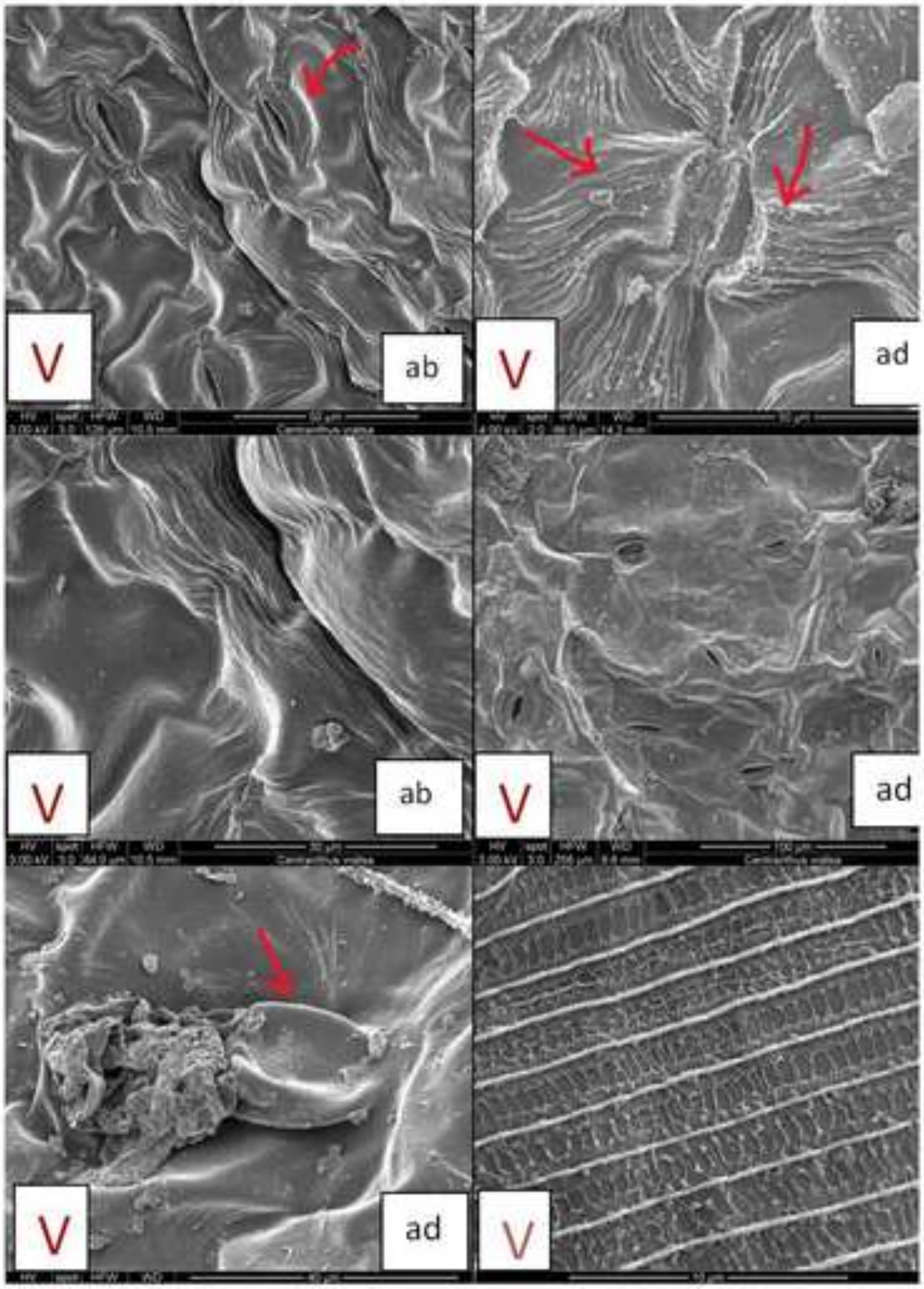

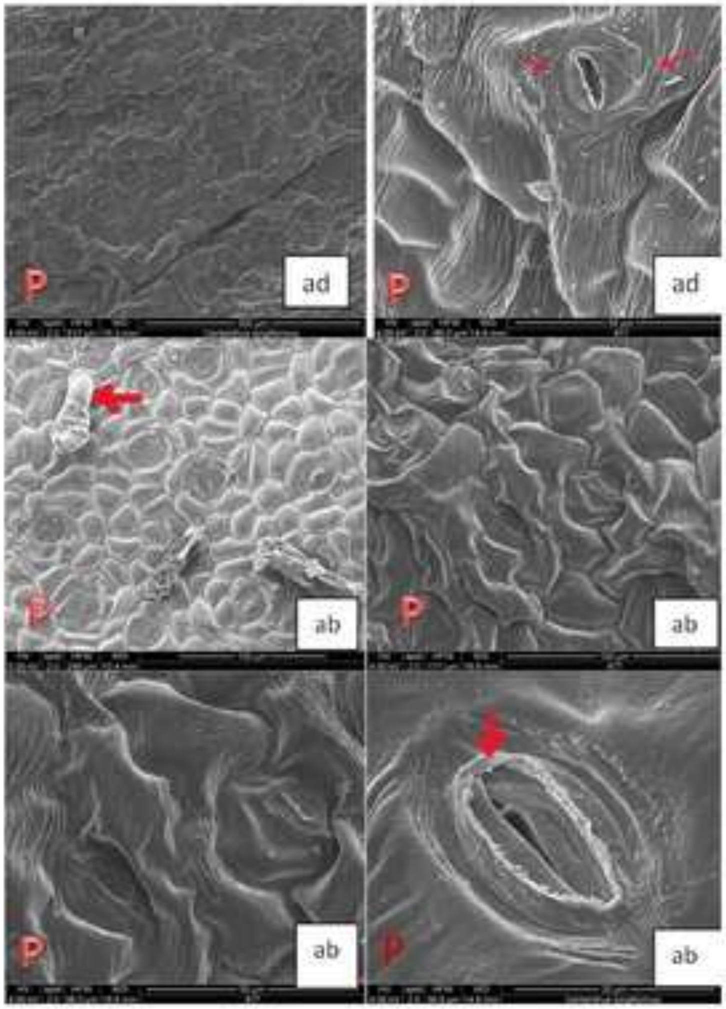
P
ad
P
ad
P
ab
P
ab
P
ab
P
ab

 Click here to access/download;Figure;Fig.9.tiff

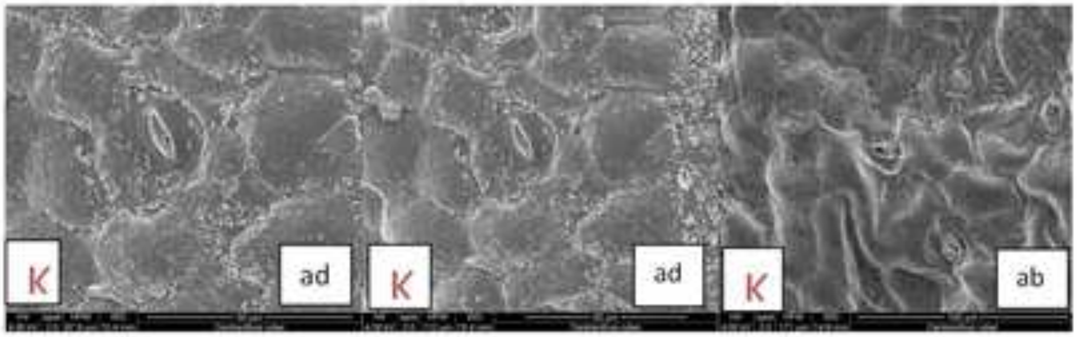

Figure10

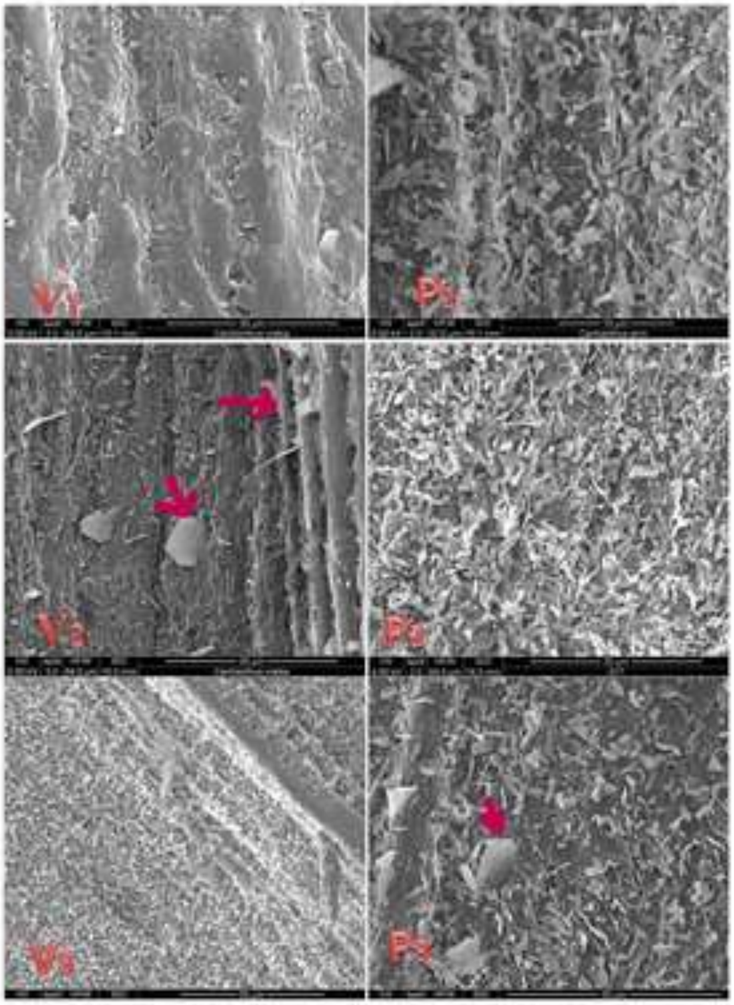

Figure11

Click here to access/download;Figure;Fig.11.tiff

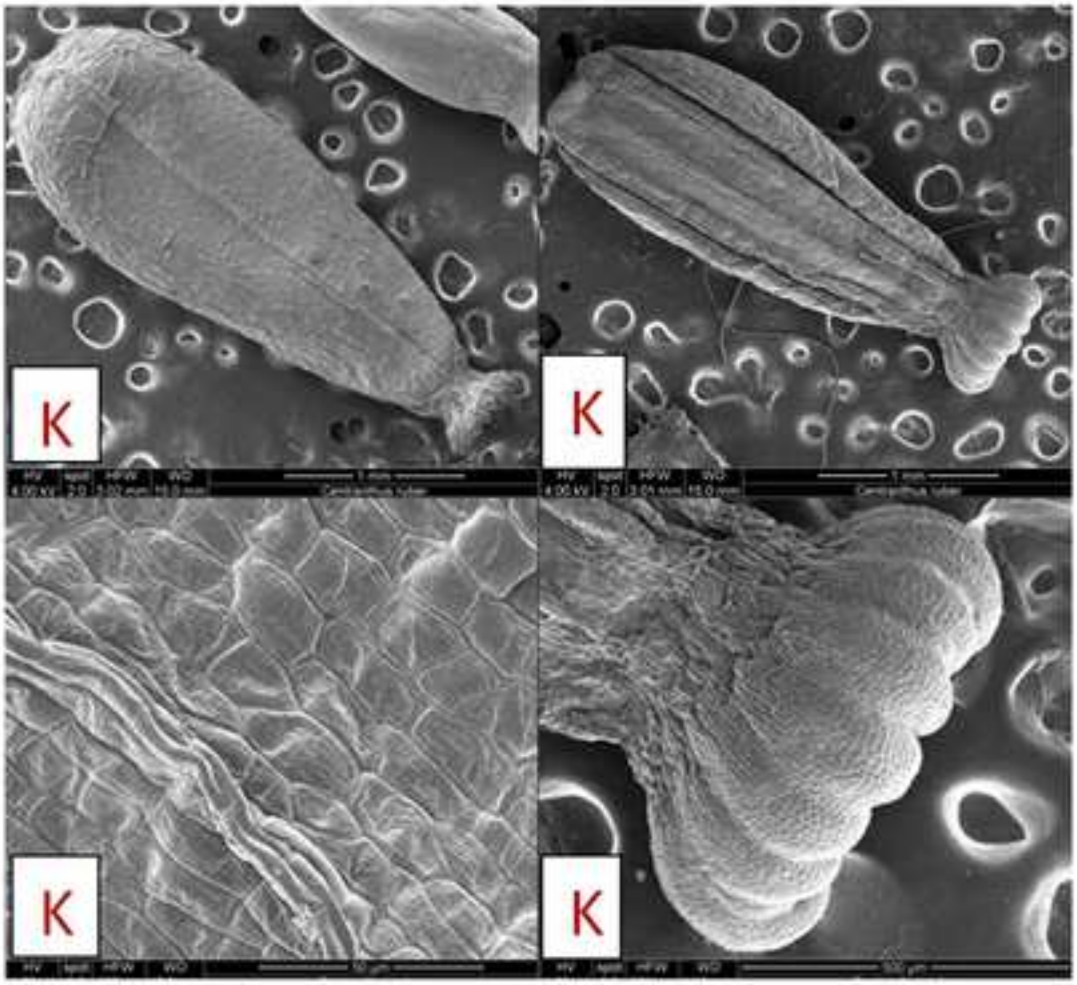

Figure12A

Click here to access/download;Figure;Fig.12A.tiff

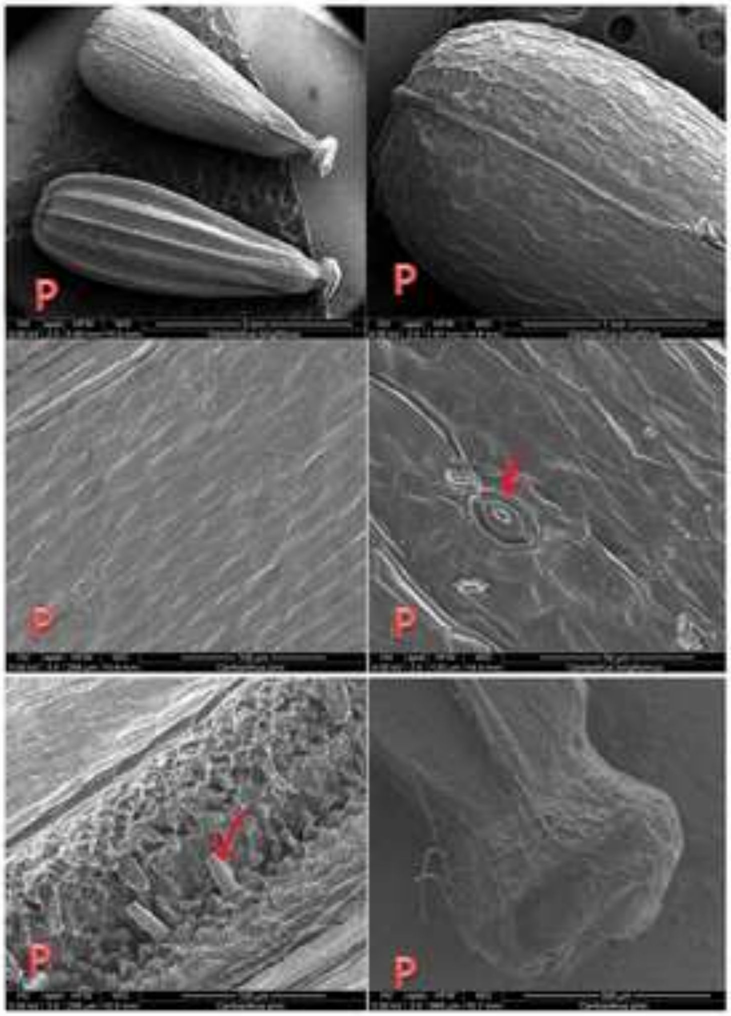

Figure12B

Click here to access/download;Figure;Fig.12B.tiff

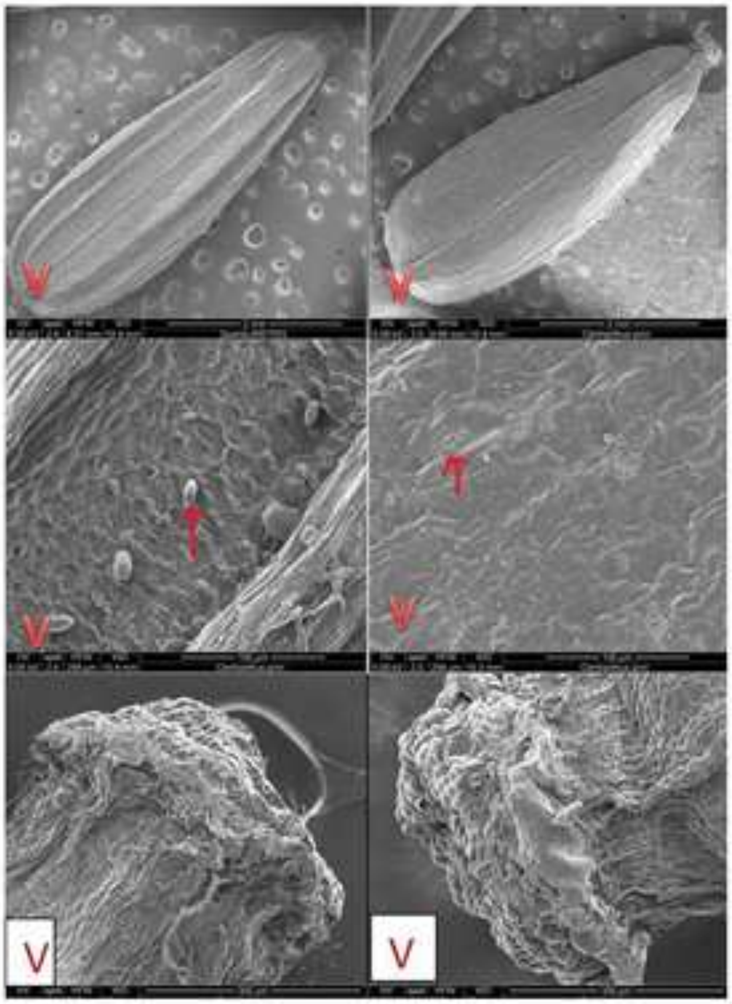

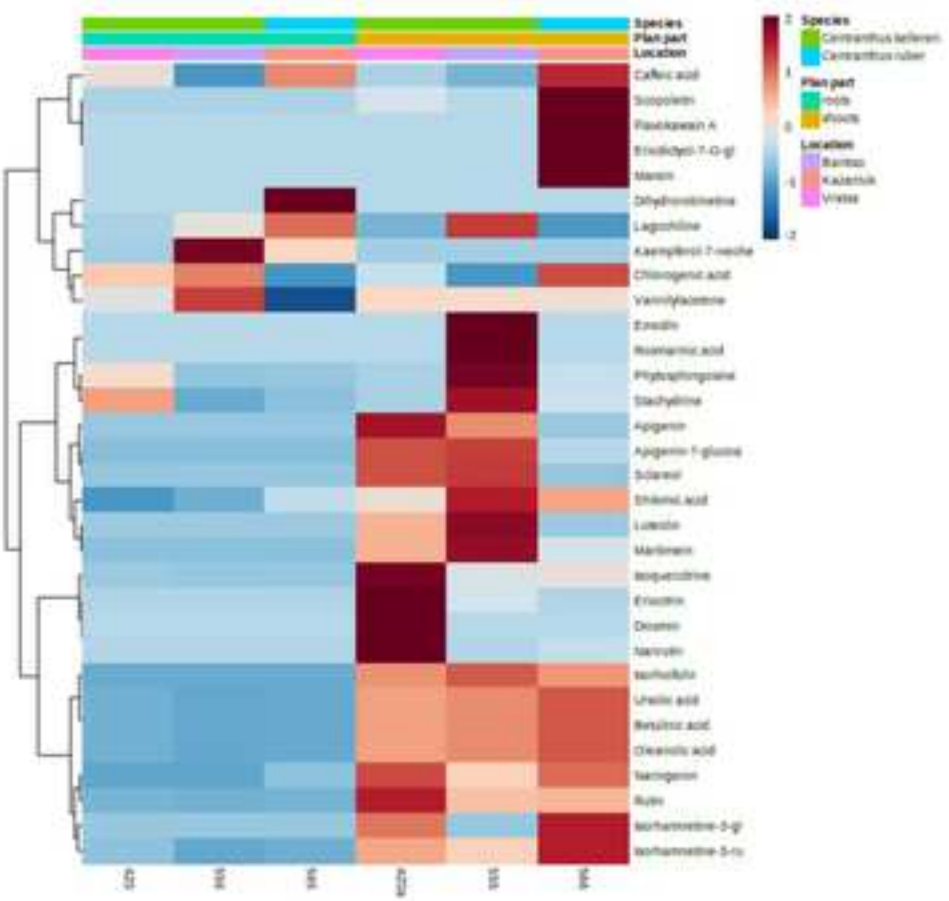
Species
Plan part
Location
Caffeic acid
Scopoletin
Eriodictyol-7-O-gl
Lagochiline
Chlorogenic acid
Vanillylacetone
Rosmarinic acid
Stachydrine
Apigenin
Apigenin 7-glucosi
Sclareol
Shikimic acid
Luteolin
Isoquercitrine
Diosmin
Narirutin
Ursolic acid
Betulinic acid
Oleanolic acid
Naringenin
Rutin
Isorhamnetine-3-gl
Isorhamnetine-3-ru
2
1
0
-1
-2
Centranthus ruber
roots
shoots
