# Supplementary material for: Phytochemical and biological investigations on Centranthus kellereri (Stoj., Stef. & T. Georgiev) Stoj. & Stef. and C. ruber (L.) DC. and their potential as new medicinal and ornamental plants
Source: PLoS One. 2023 Nov 7;18(11):e0293877. doi: 10.1371/journal.pone.0293877 (PMC10629660; doi:10.1371/journal.pone.0293877)
Supplement: Attachment — Submitted filename: Response to Reviewers_Oct.01_all.docx [file pone.0293877.s003.docx]

**Response to Reviewers**

**(Please note our responses are in bold after each of the comments)**

PONE-D-23-25651

Phytochemical and biological investigations on Centranthus kellereri (Stoj., Stef. & T. Georgiev) Stoj. & Stef. and C. ruber (L.) DC. and their potential as new medicinal and ornamental plants

PLOS ONE

Dear Dr. Zheljazkov,

Thank you for submitting your manuscript to PLOS ONE. After careful consideration, we feel that it has merit but does not fully meet PLOS ONE’s publication criteria as it currently stands. Therefore, we invite you to submit a revised version of the manuscript that addresses the points raised during the review process.

Please submit your revised manuscript by Nov 04 2023 11:59PM. If you will need more time than this to complete your revisions, please reply to this message or contact the journal office at [plosone@plos.org](mailto:plosone@plos.org). When you're ready to submit your revision, log on to <https://www.editorialmanager.com/pone/> and select the 'Submissions Needing Revision' folder to locate your manuscript file.

Please include the following items when submitting your revised manuscript:

- A rebuttal letter that responds to each point raised by the academic editor and reviewer(s). You should upload this letter as a separate file labeled 'Response to Reviewers'.
- A marked-up copy of your manuscript that highlights changes made to the original version. You should upload this as a separate file labeled 'Revised Manuscript with Track Changes'.
- An unmarked version of your revised paper without tracked changes. You should upload this as a separate file labeled 'Manuscript'.

If you would like to make changes to your financial disclosure, please include your updated statement in your cover letter. Guidelines for resubmitting your figure files are available below the reviewer comments at the end of this letter.

If applicable, we recommend that you deposit your laboratory protocols in protocols.io to enhance the reproducibility of your results. Protocols.io assigns your protocol its own identifier (DOI) so that it can be cited independently in the future. For instructions see: [https://journals.plos.org/plosone/s/submission-guidelines#loc-laboratory-protocols](https://nam04.safelinks.protection.outlook.com/?url=https%3A%2F%2Fjournals.plos.org%2Fplosone%2Fs%2Fsubmission-guidelines%23loc-laboratory-protocols&data=05%7C01%7Cvaltcho.jeliazkov%40oregonstate.edu%7C80ae7247670249de64b308dbbe3703cc%7Cce6d05e13c5e4d6287a84c4a2713c113%7C0%7C0%7C638312916299095187%7CUnknown%7CTWFpbGZsb3d8eyJWIjoiMC4wLjAwMDAiLCJQIjoiV2luMzIiLCJBTiI6Ik1haWwiLCJXVCI6Mn0%3D%7C3000%7C%7C%7C&sdata=5JM90Fvuaj%2BAr%2BS5d%2Bst5aoG0V5332u0TxWc5E99700%3D&reserved=0). Additionally, PLOS ONE offers an option for publishing peer-reviewed Lab Protocol articles, which describe protocols hosted on protocols.io. Read more information on sharing protocols at [https://plos.org/protocols?utm_medium=editorial-email&utm_source=authorletters&utm_campaign=protocols](https://nam04.safelinks.protection.outlook.com/?url=https%3A%2F%2Fplos.org%2Fprotocols%3Futm_medium%3Deditorial-email%26utm_source%3Dauthorletters%26utm_campaign%3Dprotocols&data=05%7C01%7Cvaltcho.jeliazkov%40oregonstate.edu%7C80ae7247670249de64b308dbbe3703cc%7Cce6d05e13c5e4d6287a84c4a2713c113%7C0%7C0%7C638312916299095187%7CUnknown%7CTWFpbGZsb3d8eyJWIjoiMC4wLjAwMDAiLCJQIjoiV2luMzIiLCJBTiI6Ik1haWwiLCJXVCI6Mn0%3D%7C3000%7C%7C%7C&sdata=ziPqL9czNSZhi7i5uT%2FiaJ5V0D7Xx83FCZ0lY%2FT5L9o%3D&reserved=0).

We look forward to receiving your revised manuscript.

Kind regards,

Jorddy N. Cruz
Academic Editor
PLOS ONE

Journal Requirements:

When submitting your revision, we need you to address these additional requirements.

1. Please ensure that your manuscript meets PLOS ONE's style requirements, including those for file naming. The PLOS ONE style templates can be found at 
<https://journals.plos.org/plosone/s/file?id=wjVg/PLOSOne_formatting_sample_main_body.pdf> and 
<https://journals.plos.org/plosone/s/file?id=ba62/PLOSOne_formatting_sample_title_authors_affiliations.pdf>

2. We note that you have stated that you will provide repository information for your data at acceptance. Should your manuscript be accepted for publication, we will hold it until you provide the relevant accession numbers or DOIs necessary to access your data. If you wish to make changes to your Data Availability statement, please describe these changes in your cover letter and we will update your Data Availability statement to reflect the information you provide.

Additional Editor Comments:

Dear authors,

All corrections made to the manuscript must be marked in yellow and a point-by-point answer to all questions must be provided.


[Note: HTML markup is below. Please do not edit.]

Reviewers' comments:

Reviewer's Responses to Questions

**Comments to the Author**

1. Is the manuscript technically sound, and do the data support the conclusions?

The manuscript must describe a technically sound piece of scientific research with data that supports the conclusions. Experiments must have been conducted rigorously, with appropriate controls, replication, and sample sizes. The conclusions must be drawn appropriately based on the data presented.

Reviewer #1: Yes

Reviewer #2: Yes

Reviewer #3: Yes

2. Has the statistical analysis been performed appropriately and rigorously?

Reviewer #1: Yes

Reviewer #2: Yes

Reviewer #3: Yes

3. Have the authors made all data underlying the findings in their manuscript fully available?

The [PLOS Data policy](https://nam04.safelinks.protection.outlook.com/?url=http%3A%2F%2Fwww.plosone.org%2Fstatic%2Fpolicies.action%23sharing&data=05%7C01%7Cvaltcho.jeliazkov%40oregonstate.edu%7C80ae7247670249de64b308dbbe3703cc%7Cce6d05e13c5e4d6287a84c4a2713c113%7C0%7C0%7C638312916299095187%7CUnknown%7CTWFpbGZsb3d8eyJWIjoiMC4wLjAwMDAiLCJQIjoiV2luMzIiLCJBTiI6Ik1haWwiLCJXVCI6Mn0%3D%7C3000%7C%7C%7C&sdata=2pQxdfkZA2BhgMVo7%2FDxAWPe%2B55I1JeiXyr2nq9plKw%3D&reserved=0) requires authors to make all data underlying the findings described in their manuscript fully available without restriction, with rare exception (please refer to the Data Availability Statement in the manuscript PDF file). The data should be provided as part of the manuscript or its supporting information, or deposited to a public repository. For example, in addition to summary statistics, the data points behind means, medians and variance measures should be available. If there are restrictions on publicly sharing data—e.g. participant privacy or use of data from a third party—those must be specified.

Reviewer #1: Yes

Reviewer #2: Yes

Reviewer #3: Yes

4. Is the manuscript presented in an intelligible fashion and written in standard English?

PLOS ONE does not copyedit accepted manuscripts, so the language in submitted articles must be clear, correct, and unambiguous. Any typographical or grammatical errors should be corrected at revision, so please note any specific errors here.

Reviewer #1: Yes

Reviewer #2: Yes

Reviewer #3: Yes

5. Review Comments to the Author

Please use the space provided to explain your answers to the questions above. You may also include additional comments for the author, including concerns about dual publication, research ethics, or publication ethics. (Please upload your review as an attachment if it exceeds 20,000 characters)

Reviewer #1: Many correction in text.

**Response:** **Because most notes relate to citing authors, we have inserted authors where necessary.**
1. Abstract clear but some corrections'

**Response**: **We correct all plant name in Italic.**

2. Introduction has to long and many corrections in text.
**Response**: **We carefully checked the text and made corrections as necessary. Also, we have shortened the introduction to make the article easier to read.**

3. Material and methods have manty corrections in text.
**Response**: **We have shortened the part of section M&M so that the methods are repeatable and the paper is easy to read.**

Results have many corrections in text.
**Response**: **We corrected the Latin name of the species and cited authors as suggested.**

5. Discussion is clear but it has many corrections in text.
**Response**: **We corrected the Latin name of the species and cited authors as suggested.**

6. Conclusion is clear
**Response**: **Thank you**

7. References please check again follow the journal
**Response**: **We checked all authors. They are formatted according to the Journals requirements.**

Good luck

Reviewer #2: This manuscript entitled “Phytochemical and biological investigations on Centranthus kellereri (Stoj., Stef. & T. Georgiev) Stoj. & Stef. and C. ruber (L.) DC. and their potential as new medicinal and ornamental plants” described phytochemical, microstructural differences, and genetic differences of C. kellereri individuals from the two locations and biological activity of . The manuscript had the interesting subject and I think the manuscript is good for publication in this journal

**Response**:**Thank you**!

Reviewer #3: The manuscript makes important contributions to the phytochemical and botanical knowledge of the species studied. It is a well-structured article, with cohesive ideas and well-presented arguments that effectively validate the results obtained. Here are some recommendations for improving the manuscript.

1) The introduction contains a lot of relevant information about the species and the importance of the study, but it is very long and makes for tiresome reading. I suggest that the author be more succinct in the introduction of the manuscript.
**Response**: **We have shortened the introduction to make the article easier to read.**

2) In line 110, the author says that "The closely related species Centranthus ruber DC has been extensively studied, with several reports on its bioactivity and the potential health benefits of its constituents". Reading the passage marked in red gives the impression that the plant offers benefits to its constituents and not to humans. I suggest that the author rewrite the passage to remove the ambiguity.
**Response**:**We rewrite this sentence as follows: “The phytochemical composition of C. ruber has been extensively studied [7-11]. There were several reports on its compositions, bioactivity and potential health benefits [7-11].**

3) The methodology of the manuscript contains many details, including the preparation of the solutions. In my opinion, there is no need to describe the preparation of the reagents, since the methods used in the article are very well described in the literature. I recommend removing the description of the preparation of the reagents to make the methodology more summarized and more pleasant to read.
**Response**: **Thank you! We shortened methodology where described the preparation of the reagents as suggested.**

4) I noticed that (2E,6E), n-hexadecoic acid was identified in the EO extracted with hexane. I suggest revising the identification of the sample, as it is unlikely that the plant can synthesize a fatty acid with trans stereochemistry.
**Response**: **Thanks you very much for your comment. In agreement with this comment, we deleted “(E,E)-“ and left only “Geranyl linalool” in the Table and throughout the text.**

**Also, you noticed this: “Table 9. Chemical profile of the hexane-extracted samples: Mean concentration (%) of 3-methylbutanoic acid (isovaleric acid) (3-Meth-Iso), 3-methylpentanoic acid (3-methylvaleric acid) (3-Meth-3-M), (2E)-tridecen-1-al (2E), n-tetradecanal (n-Tetrad), (2E,6E)-farnesyl acetate (2E,6E), n-hexadecoic acid (n-Hex), and (Z,Z)-9,12-octadecadienoic acid (Z,Z)”, in response, perhaps there is a confusion with the fact that these are two different compounds: (2E,6E)-farnesyl acetate (2E,6E) and n-hexadecoic acid (n-Hex). The name of the acid is misspelled. It should be read n-hexadecanoic acid (n-Hex). It is actually palmitic acid. 5) Lines 434 and 470 and tables 5 and 8 contain the term "sesqoiterpenoids". The correct spelling is "sesquiterpenoids".
Response**: **Corrected**

6) The pictures are of poor quality, I suggest improving them and resending them.

**Response**: **We improved the pictures of the embryological part of this research.**

6. PLOS authors have the option to publish the peer review history of their article ([what does this mean?](https://nam04.safelinks.protection.outlook.com/?url=https%3A%2F%2Fjournals.plos.org%2Fplosone%2Fs%2Feditorial-and-peer-review-process%23loc-peer-review-history&data=05%7C01%7Cvaltcho.jeliazkov%40oregonstate.edu%7C80ae7247670249de64b308dbbe3703cc%7Cce6d05e13c5e4d6287a84c4a2713c113%7C0%7C0%7C638312916299095187%7CUnknown%7CTWFpbGZsb3d8eyJWIjoiMC4wLjAwMDAiLCJQIjoiV2luMzIiLCJBTiI6Ik1haWwiLCJXVCI6Mn0%3D%7C3000%7C%7C%7C&sdata=7E%2FSLXJWWshPK%2BDGQdwO8%2FP3Uo%2BO03WU8Ys4MfjarFc%3D&reserved=0)). If published, this will include your full peer review and any attached files.


If you choose “no”, your identity will remain anonymous but your review may still be made public.


**Do you want your identity to be public for this peer review?** For information about this choice, including consent withdrawal, please see our [Privacy Policy](https://nam04.safelinks.protection.outlook.com/?url=https%3A%2F%2Fwww.plos.org%2Fprivacy-policy&data=05%7C01%7Cvaltcho.jeliazkov%40oregonstate.edu%7C80ae7247670249de64b308dbbe3703cc%7Cce6d05e13c5e4d6287a84c4a2713c113%7C0%7C0%7C638312916299095187%7CUnknown%7CTWFpbGZsb3d8eyJWIjoiMC4wLjAwMDAiLCJQIjoiV2luMzIiLCJBTiI6Ik1haWwiLCJXVCI6Mn0%3D%7C3000%7C%7C%7C&sdata=1p%2BH6BWOlHekMLg1pxLQt926RyiXnhxGleL9Uvoofg8%3D&reserved=0).

Reviewer #1: No

Reviewer #2: **Yes:** Dr. Mohammad Moghaddam

Reviewer #3: No

[NOTE: If reviewer comments were submitted as an attachment file, they will be attached to this email and accessible via the submission site. Please log into your account, locate the manuscript record, and check for the action link "View Attachments". If this link does not appear, there are no attachment files.]

While revising your submission, please upload your figure files to the Preflight Analysis and Conversion Engine (PACE) digital diagnostic tool, [https://pacev2.apexcovantage.com/](https://nam04.safelinks.protection.outlook.com/?url=https%3A%2F%2Fpacev2.apexcovantage.com%2F&data=05%7C01%7Cvaltcho.jeliazkov%40oregonstate.edu%7C80ae7247670249de64b308dbbe3703cc%7Cce6d05e13c5e4d6287a84c4a2713c113%7C0%7C0%7C638312916299095187%7CUnknown%7CTWFpbGZsb3d8eyJWIjoiMC4wLjAwMDAiLCJQIjoiV2luMzIiLCJBTiI6Ik1haWwiLCJXVCI6Mn0%3D%7C3000%7C%7C%7C&sdata=7TzjkpH0oQOojogQ00ttDlR9RBCdPxLQZEvWZcAmwXc%3D&reserved=0). PACE helps ensure that figures meet PLOS requirements. To use PACE, you must first register as a user. Registration is free. Then, login and navigate to the UPLOAD tab, where you will find detailed instructions on how to use the tool. If you encounter any issues or have any questions when using PACE, please email PLOS at [figures@plos.org](mailto:figures@plos.org). Please note that Supporting Information files do not need this step.
